# Effect of Antioxidant-Rich Diets, Rearing, Harvesting, and Processing Conditions on Off-Odor Volatile Formation, Lipid Content, Fatty Acid Profile, and Antioxidant Activity in Yellow Perch (*Perca flavescens*)

**DOI:** 10.3390/foods15183257

**Published:** 2026-09-15

**Authors:** Manpreet Kaur, Md Zakir Hossain, Peter Shep, Alexander Gregory, Rachel M. Cole, Dong-Fang Deng, Sheryl Barringer

**Affiliations:** 1 Department of Food Science and Technology, The Ohio State University, Columbus, OH 43210, USA; kaur.333@osu.edu (M.K.); hossain.154@osu.edu (M.Z.H.); cole.311@osu.edu (R.M.C.); 2 School of Freshwater Sciences, University of Wisconsin-Milwaukee, Milwaukee, WI 53204, USA; shep@uwm.edu (P.S.); gregor75@uwm.edu (A.G.); dengd@uwm.edu (D.-F.D.)

**Keywords:** yellow perch, off-odor, volatile compounds, SIFT-MS, antioxidant diet, lipid oxidation, fatty acid profile, protein degradation, geosmin, 2-methylisoborneol, aquaculture, antioxidant capacity, vitamin E, tissue distribution

## Abstract

Yellow perch (*Perca flavescens*) is a high-value aquaculture species, but variation in fillet nutritional composition and off-odor formation can limit product quality. This study evaluated how diets with plant-based antioxidants, rearing environment, use of heat shock, post-harvest processing, and fish weight affected off-odor volatile formation, lipid content, fatty acid profile, antioxidant activity, and vitamin E content in yellow perch fillets. Fish were fed camu camu, wheat flour, mixed starch, or commercial diets for 9 weeks, harvested with or without heat shock, and analyzed after frozen and refrigerated storage or baking. Volatiles were measured by selected-ion flow-tube mass spectrometry, while lipid content, antioxidant activity, vitamin E, and fatty acid profile were evaluated to characterize fillet composition and oxidative stability. Under standard harvest conditions, camu camu– and wheat-fed fish generally showed lower concentrations of lipid oxidation– and protein degradation–derived volatiles than mixed starch- and commercial-fed fish, especially after refrigerated storage or baking. These volatile differences were supported by differences in fillet composition and antioxidant status, with camu camu–fed fish showing the highest lipid content, while camu camu– and wheat-fed fish showed higher antioxidant activity than mixed starch-fed fish. Fatty acid profiling provided additional information on lipid composition and its potential relationship with lipid oxidation–derived off-odor formation. Heat shock increased volatile formation and reduced diet-dependent differences in the volatile profile. Heat shock also increased antioxidant activity across diets, eliminating diet-related differences in antioxidant activity. Rearing water systems and fish weight had limited effects on the volatile profile. These findings show that plant-based antioxidant diets can influence both nutritional quality and off-odor stability in yellow perch, particularly when combined with low-stress harvest and appropriate post-harvest handling.

## 1. Introduction

Yellow perch (*Perca flavescens*) is an important freshwater aquaculture species valued for its mild flavor, firm texture, and market potential [1]. However, the development of undesirable odors can reduce fillet quality and consumer acceptance [2,3]. Volatile formation in fish is complex because compounds can originate from lipid oxidation, protein degradation, microbial activity, environmental exposure, and thermal reactions during cooking. While cooking can generate desirable cooked aromas, it can also increase the release or formation of compounds associated with rancid, sulfurous, fishy, earthy, or musty notes when lipid oxidation, protein degradation, or environmental off-flavor compounds are present [2,3,4]. Understanding how pre-harvest and post-harvest factors influence these volatile compounds is therefore important for improving yellow perch flavor quality [1,5].

Lipid oxidation is one of the major pathways responsible for off-odor formation in fish, even in lean fish [3,4]. Because fish lipids are rich in unsaturated fatty acids, they are susceptible to oxidation during storage and cooking [2,3]. Oxidative degradation of lipids produces aldehydes, alcohols, ketones, hydrocarbons, and furans that are associated with rancid, grassy, fatty, and fishy aromas [2,3,4]. Protein degradation can also contribute to off-odor formation through the production of sulfur-containing compounds, amines, aldehydes, and other nitrogen-containing volatiles [3,6,7]. These compounds may form through endogenous enzymatic activity, microbial metabolism, and thermal reactions involving amino acids and other precursors [3,7,8].

In addition to lipid- and protein-derived volatiles, environmental off-flavor compounds are a major concern in freshwater aquaculture [1,3,9]. Compounds such as geosmin and 2-methylisoborneol are produced by certain microorganisms in aquatic environments and can be absorbed by fish from the surrounding water [9,10,11]. Because these compounds are lipophilic, they can partition into fish tissue and persist even after harvest [5,9,10]. Their earthy and musty aromas can be detected at low concentrations, making them important contributors to consumer rejection [10,11,12]. Control of these compounds depends strongly on water quality management, system design, microbial ecology, and effective depuration practices [1,10,13].

Dietary formulation is one potential strategy for reducing off-odor development in aquaculture fish [1,4]. Antioxidants such as vitamin C, tocopherols, phenolic compounds, and polyphenols can limit oxidative reactions by scavenging radicals, chelating pro-oxidant metals, or interrupting lipid radical propagation [4,7]. However, antioxidant effectiveness depends on chemical structure, tissue distribution, solubility, thermal stability, and interaction with lipid and protein substrates [4]. Therefore, different dietary antioxidant sources may provide different levels of protection during storage and cooking [1,4].

Fish muscle lipid content and fatty acid composition are additional determinants of both nutritional quality and susceptibility to lipid oxidation–derived off-odor formation. Yellow perch fillets are relatively lean but contain a substantial proportion of polyunsaturated fatty acids, which are more susceptible to oxidative degradation than saturated or monounsaturated fatty acids [14,15]. Dietary fatty acid composition can influence the fatty acid profile deposited in fish muscle, and diets that alter the ratio of polyunsaturated to saturated fatty acids may therefore affect both lipid oxidation susceptibility and the resulting volatile profile [14]. Because fatty acid profile and lipid content can vary with diet formulation and ingredient source, evaluating both alongside antioxidant activity provides a more complete picture of how diet influences fillet quality and off-odor stability.

Heat shock may also influence volatile formation. Acute stress before harvest can alter fish physiology, increase oxidative stress, and change antioxidant defense responses [5,16]. Elevated temperature may also affect membrane permeability, metabolism, and the release or redistribution of lipophilic compounds [5,17]. However, it is unclear whether short-term heat exposure before harvest can reduce environmental off-flavor compounds or whether it instead alters tissue chemistry in ways that affect volatile formation during storage and cooking [9,16].

Other production factors, including rearing water system and fish size, may also contribute to flavor quality. Rearing systems differ in water exchange, microbial communities, water quality, and potential exposure to environmental off-flavor compounds [4,5,17]. Fish weight may influence volatile formation through changes in tissue composition, lipid content, and growth stage. However, the relative importance of these factors compared with diet, harvest condition, and post-harvest handling remains unclear for yellow perch.

Therefore, this study aimed to evaluate how pre-harvest factors, including antioxidant-diet formulation, use of heat shock, rearing water system, and fish weight, influence off-odor volatile formation in yellow perch fillets. To help explain the mechanisms underlying diet- and condition-related differences in volatile formation, antioxidant activity, lipid content, and vitamin E content of the fillet were also measured, along with antioxidant activity and total volatile content in liver and visceral fat tissue, to evaluate how dietary antioxidants and lipid substrate were distributed across tissues and whether tissue-level composition corresponded to fillet-level volatile outcomes. Fillets were evaluated after frozen storage, refrigerated storage, and baking to determine how post-harvest handling affected diet-related volatile formation. Headspace volatile compounds were measured using selected-ion flow-tube mass spectrometry and grouped as lipid oxidation volatiles, protein degradation volatiles, and environmental off-flavor compounds. The overall goal was to identify factors contributing to yellow perch off-odor formation and to provide guidance for improving flavor quality in aquaculture production.

## 2. Materials and Methods

### 2.1. Effect of Antioxidant-Diet Formulation and Harvest Conditions

#### 2.1.1. Fish Rearing and Feeding

Yellow perch were reared at the University of Wisconsin–Milwaukee Deng laboratory facility. A 9-week feeding trial was conducted using juvenile yellow perch (*Perca flavescens*) with an initial average body weight of 5.0 g. Fish were randomly distributed into 12 tanks (120 L capacity) at a stocking density of 20 fish per tank. The tanks were connected to a flow-through aquaculture system with a constant flow rate of 3 L min^−1^. The four dietary treatments used in this study consisted of one control diet (wheat flour), two experimental diets (camu camu and mixed starch), and one commercial reference diet. There were 3 tanks per diet, and each tank was considered a replicate. Fish were fed four times daily (09:00, 12:00, 15:00, and 18:00) at an initial feeding rate of 3–4% of their body weight per day, anticipating an estimated feed conversion ratio (FCR) of 1.0. Every two weeks, fish were group-weighed, and the feeding rate was adjusted accordingly based on the new biomass. Optimal water quality parameters were maintained throughout the trial to promote growth: temperature (22–24 °C), dissolved oxygen (>8 mg L^−1^), pH (7.5–8.0), and total ammonia nitrogen (<0.01 mg L^−1^). A 12-h light and 12-h dark (12L:12D) photoperiod was maintained. All animal handling and rearing procedures were conducted under an approved animal protocol by the Animal Care and Use Committee of the University of Wisconsin–Milwaukee (Protocol Number 21-22#32, approved on 18 March 2024).

#### 2.1.2. Experimental Diets

Three experimental diets (Table 1) were formulated to contain approximately 50% crude protein and 12% crude lipid. The control diet utilized 15% wheat flour as the primary carbohydrate source. In the camu camu diet, wheat flour was replaced with camu camu (*Myrciaria dubia*). The mixed starch diet replaced the wheat flour with a mixed starch blend, comprising equal proportions of wheat, corn, and tapioca starch. All experimental diets were manufactured using a cold-extrusion method at the University of Wisconsin–Milwaukee (UWM) and stored at –20 °C until needed. A commercial feed (Zeigler starter feed (1.5 mm), Gardners, PA, USA) containing 50% crude protein and 15% crude lipid was included as a reference diet.

#### 2.1.3. Harvest Conditions—With and Without Heat Shock Treatment

Yellow perch were harvested either with or without heat shock treatment. For the harvest without heat shock, fish were maintained at the normal rearing temperature of 22 °C before sampling. For the heat shock treatment, fish remained in the flow-through aquaculture system with continuous water exchange at 3 L min^−1^, and the water temperature was increased from 21.6 to 30.6 °C at a rate of 1 °C every twenty minutes, then held at 30.6 °C for 18 h before sampling. Fish were euthanized with an overdose of MS222 and stored at −80 °C until shipping in a Styrofoam box surrounded by dry ice and shipped overnight to The Ohio State University. Upon arrival, samples were immediately stored at −80 °C until further processing. Samples were collected from yellow perch fed wheat, camu camu, mixed starch, or commercial diets before or after heat shock treatment. For volatile analysis, frozen samples were thawed for 30 min before sample preparation.

#### 2.1.4. Harvest, Storage and Cooking

After harvest, fillets were assigned to frozen, refrigerated, or baked treatments. Frozen samples were stored at −80 °C for 2 months and thawed for 30 min on ice before analysis. Refrigerated samples were held at 4 °C for 3 days before analysis. Baked samples were first held at 4 °C for 3 days and then baked at 200 °C for 15 min before volatile analysis. Fillets were analyzed for headspace volatile compounds using the SIFT-MS method.

### 2.2. Effect of Rearing Water Systems

To evaluate the effect of rearing water system on off-odor volatile formation, yellow perch fillets were obtained from five sources: recirculating aquaculture system RAS 1 (CJ Fish Company, OH, USA), recirculating aquaculture system RAS 2 (The Ohio State University, Columbus, OH, USA), flow-through system FTS (fish from the commercial diet group in the feeding trial described in Section 2.1.1), pond-reared fish (The Ohio State University South Centers, Piketon, OH, USA), and wild-caught fish (Lake Erie, OH, USA). The wild fish were included as a natural reference for comparison with aquaculture-produced fish. Because fish from different sources were not reared under a single controlled diet, age, genetic background, harvest history, or management protocol, this comparison was interpreted as an observational comparison of production sources rather than a controlled test of the rearing water system alone. Fillets from each source were analyzed using the same SIFT-MS volatile analysis headspace method.

### 2.3. Effect of Fish Weight

To evaluate whether fish size affected off-odor volatile formation, yellow perch were reared in the same RAS system for two to five years. Fish in this comparison were raised under the same diet, rearing system, and management conditions, and were sorted by body weight at harvest, allowing the effect of body weight to be evaluated without the additional diet and system-level differences present in the rearing system comparison. Fillets from each weight group were collected and analyzed for headspace volatile compounds using the SIFT-MS method.

### 2.4. Selected-Ion Flow-Tube Mass Spectrometry Headspace Analysis

Headspace volatile compounds in yellow perch fillets were analyzed using selected-ion flow-tube mass spectrometry (SIFT-MS), following the method used in our previous fish study [5]. Prior to SIFT-MS analysis, a 2 g portion of minced muscle tissue from each fillet was transferred to a 500 mL Pyrex bottle. To facilitate volatile release, 20 mL of 0.5% (*v*/*v*) ethanol was added, and the bottles were sealed with open-top, septum-lined caps. Samples were homogenized using a vortex mixer for 1 min and equilibrated in a water bath at 42 °C for 30 min. Headspace volatile compounds were analyzed using SIFT-MS (Voice200ultra, Syft Technologies, Christchurch, New Zealand). Analyses were performed in selected-ion monitoring mode using H_3_O^+^, NO^+^, and O_2_^+^ precursor ions. The volatile compounds quantified in this study were the same as those measured in our previous study on salmon [5]. Compound concentrations were calculated using established ion-molecule reaction rate coefficients. Instrument calibration was verified using a certified gas standard containing benzene, ethylbenzene, toluene, and xylene isomers prior to sample analysis. Headspace sampling was conducted using a 14-gauge passivated needle, with the inlet temperature maintained at 175 °C. Each sample was analyzed over a 120 s acquisition period. Three analytical replicates were performed per sample type. An empty Pyrex bottle was used as a blank.

### 2.5. Muscle (Fillet), White Muscle, Liver, and Visceral Fat—Lipid Content, Antioxidant Activity, and Vitamin E Analysis

#### 2.5.1. Lipid Content

Total lipids were extracted from fish tissue, methylated, and quantified by gas chromatography with flame ionization detection (GC-FID) [18,19,20]. Briefly, 1.5 g of tissue was combined with 20 mL of a chloroform-methanol (2:1, *v*/*v*) mixture and homogenized for 1–2 min. The homogenate was filtered under vacuum, and 4 mL of saturated MgCl_2_·6H_2_O solution was added to facilitate phase separation. The lipid layer was collected, evaporated under nitrogen with mild heating, and the total lipid content was recorded.

#### 2.5.2. Methylation of Fatty Acid Derivatives

1.5 mL of methanolic sodium hydroxide was added to the sample, and the mixture was incubated at 80 °C for 1 h. After that, 2 mL of boron trifluoride (BF_3_) in methanol was added to both NL and PL fractions, and the samples were incubated at 80 °C for an additional 30 min. After cooling, 1 mL of hexane and 1 mL of distilled water were added to each sample and vortexed for 1 min. The upper hexane phase, containing the fatty acid methyl esters (FAMEs), was collected and passed through anhydrous sodium sulfate to remove residual moisture and impurities. The FAMEs were analyzed utilizing a gas chromatograph equipped with a flame ionization detector and a 30-m Omegawax 320 fused silica capillary column (Supelco, Bellefonte, PA, USA), using helium as the carrier gas. The oven temperature program was initiated at 175 °C, ramped at 3 °C/min to 220 °C, and then decreased at 5 °C/min to a final temperature of 210 °C. Peak identification was performed by comparing sample retention times against a commercial FAME standard mixture (Matreya, LLC, Pleasant Gap, PA, USA). Fatty acid profiles are reported as a percentage of total identified fatty acids, consistent with previously established protocols [21,22,23].

#### 2.5.3. Antioxidant Activity

Antioxidant activity was determined using the ABTS (2,2′-azino-bis (3-ethylbenzothiazoline-6-sulfonic acid)) radical scavenging assay (RSA), with slight modifications [14,24]. Antioxidant compounds were extracted by homogenizing 1 g of tissue in 7 mL of hexane/ethanol (5:2, *v*/*v*), followed by centrifugation at 4000 rpm for 5 min; the ethanol phase was retained for the assay. The ABTS radical cation (ABTS·^+^) was generated by mixing 7 mM ABTS with 2.45 mM potassium persulfate and allowing the reaction to proceed in the dark for 16 h, then diluted to an absorbance of ~1.1 at 734 nm. For the assay, 2 mL of diluted ABTS·^+^ was mixed with 100 µL of sample, incubated in the dark for 30 min, and absorbance was measured at 734 nm. The same extraction and assay procedure was applied to white muscle, liver, and visceral fat tissue to evaluate tissue-specific antioxidant distribution (Section 3.5).

#### 2.5.4. Vitamin E Analysis

Vitamin E (alpha-tocopherol) content was measured by HPLC [25]. Approximately 1 g (±50 mg) of frozen fillet was homogenized in 1 mL of methanol containing 0.1% BHT, then extracted twice with a 1:1 (*v*/*v*) hexane:acetone mixture following phase separation with cold saturated NaCl. The pooled non-polar phase was evaporated under argon, and the dried residue was redissolved in MTBE:methanol (1:1) for analysis on a high-performance liquid chromatography with diode-array detection (HPLC-DAD) system using a ZORBAX Stable Bond SB-C18 column (100 × 4.6 mm, 3.5 µm) with gradient elution.

### 2.6. Statistical Analysis

Statistical analyses were conducted using JMP^®^ Pro Version 16.0.0 (SAS Institute Inc., Cary, NC, USA). Figures were generated using MATLAB^®^ R2024b Update 5 (MathWorks, Natick, MA, USA). Volatile compounds were analyzed using analysis of variance (ANOVA) appropriate to each experimental design. For the diet and harvest-condition study, total volatile content and volatile category totals were analyzed using a three-way factorial ANOVA. The fixed factors were diet, harvest condition, and storage or cooking treatment. The model included the main effects, all two-way interactions, and the three-way interaction among diet, harvest condition, and storage or cooking treatment. Individual volatile compounds were analyzed separately within each harvest condition and storage or cooking treatment using one-way ANOVA to compare the four diets. For the rearing water system comparison, one-way ANOVA was used with rearing system as the fixed factor. For the fish weight comparison, one-way ANOVA was used with fish weight as the fixed factor. When significant differences were detected, Fisher’s least significant difference test was used for mean separation. Differences were considered significant at *p* < 0.05. Different letters in tables and figures indicate significant differences among treatments within each volatile compound. Volatile concentrations were plotted on a base-10 logarithmic scale for visualization. For the diet and harvest-condition comparisons, each diet was represented by 3 tanks, with one fish sampled per tank for each harvest condition (with and without heat shock), giving *n* = 3 independent replicates per diet within each harvest condition, and 6 fish per diet in total across both harvest conditions. Muscle (fillet), liver, and visceral fat lipid content, antioxidant activity (ABTS), and vitamin E content were each analyzed using the one-way ANOVA (diet, harvest condition, rearing environment, or tissue as the fixed factor, as appropriate), with Tukey’s Honestly Significant Difference (HSD) post hoc test applied for multiple mean comparisons (*p* < 0.05), using JMP Student Edition 18 (JMP Statistical Discovery LLC, Cary, NC, USA). Total volatile content in visceral fat and white muscle was analyzed separately by one-way ANOVA with diet as the fixed factor within each tissue, using the same significance threshold (*p* < 0.05). Fisher’s least significant difference test was used for the volatile compound dataset because it involved a large number of individually screened compounds across multiple experimental designs, where a less conservative mean-separation test was considered appropriate for exploratory compound-by-compound comparisons; Tukey’s HSD was applied to the lipid, antioxidant, vitamin E, and tissue datasets because these involved fewer, targeted comparisons per response variable, where a more conservative correction for multiple comparisons was warranted.

## 3. Results and Discussion

This study evaluated the effect of four dietary antioxidant strategies on the volatile profile of yellow perch fillets reared at 22 °C without heat shock, versus after acute heat shock at 30 °C for 18 h, followed by three storage/processing conditions (frozen, refrigerated, and baked). The diets differed in ingredient sources and were designed to provide distinct antioxidant systems that may influence off-odor volatile formation. The camu camu diet contained camu camu powder as a source of vitamin C and fruit-derived phenolics, including ellagic acid, ellagitannins, gallic acid derivatives, flavonols, flavanols, anthocyanins, and proanthocyanidins [4,26,27,28]. The wheat diet contained wheat flour as a source of cereal-derived phenolic acids, primarily ferulic acid, with contributions from p-coumaric, sinapic, caffeic, vanillic, and syringic acids [29,30,31,32], as well as lipid-associated antioxidants such as tocopherols [33]. The mixed starch diet contained a blend of wheat, corn, and tapioca starches with limited antioxidant contribution and the basal vitamin mix, which includes vitamin C, and the commercial diet served as a reference feed. Therefore, differences in off-odor volatiles were interpreted in relation to antioxidant type, antioxidant activity, lipid substrate availability, and feed composition.

Off-odor volatile formation in yellow perch fillets was significantly affected by diet for lipid oxidation volatiles (*p* = 0.0007) and protein degradation volatiles (*p* < 0.0001), but not environmental contaminant volatiles (*p* > 0.05). Use of heat shock at harvest significantly affected lipid oxidation volatiles (*p* = 0.0027), protein degradation volatiles (*p* < 0.0001), and environmental contaminant volatiles (*p* < 0.0001). Storage or cooking also significantly affected all volatile categories (*p* < 0.0001). Significant diet × heat shock interactions (*p* < 0.0015) were observed for all volatile groups, indicating that diet effects differed with and without heat shock. Significant heat shock × storage or cooking interactions (*p* < 0.0001) were also observed for all volatile groups, indicating that the effect of storage or cooking depended on the use of heat shock at harvest.

### 3.1. Effect of Diets and Post-Harvest Treatment on the Quality of Yellow Perch Fillets

**Figure 1:**
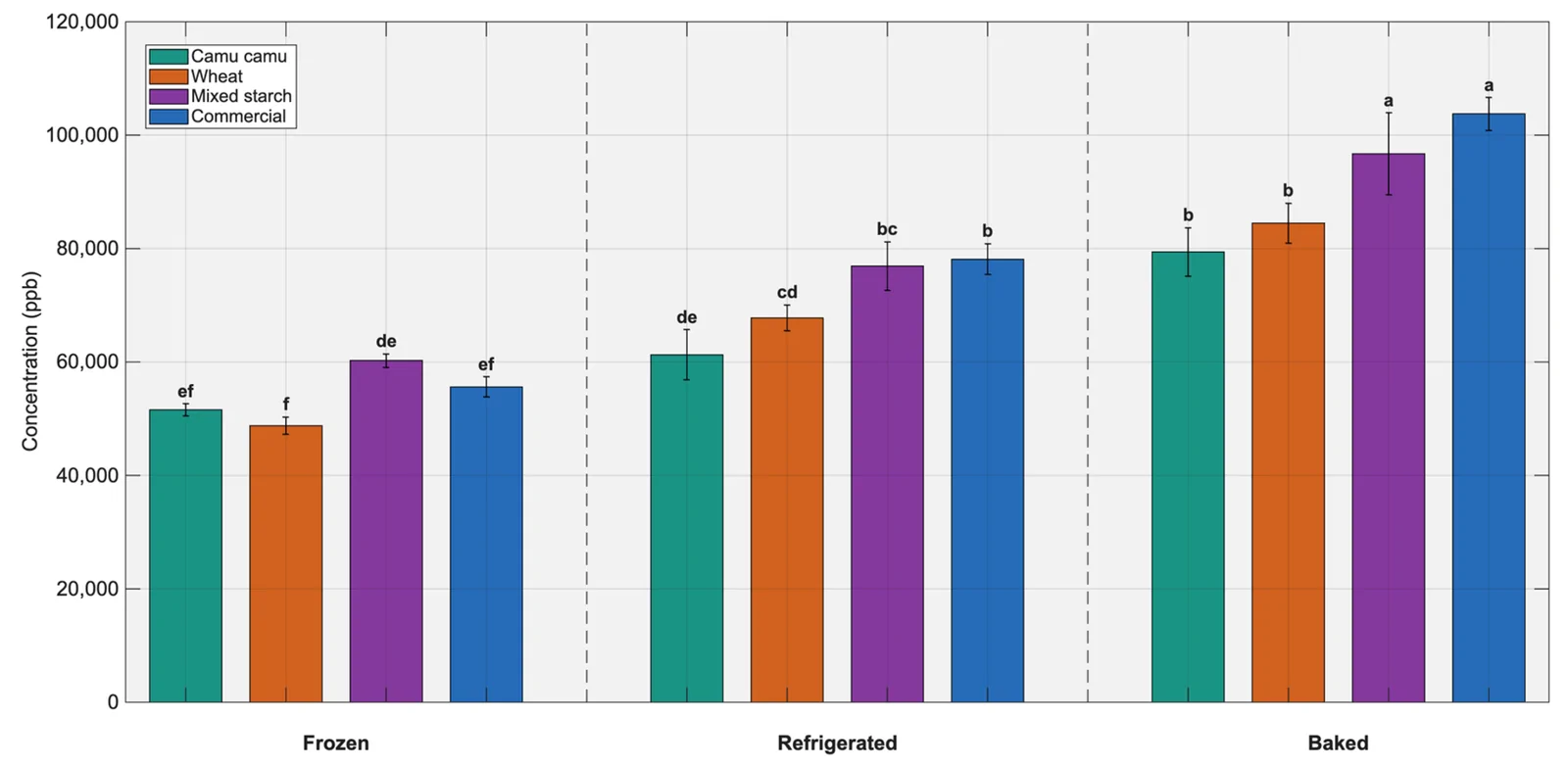
Effect of antioxidant-diet formulation on the total volatile content in frozen, refrigerated, and baked yellow perch fillets harvested without heat shock conditions. Values are means (n = 3 per treatment). Different letters indicate significant differences among the four diets within each combination of harvest condition and storage/cooking treatment (*p* < 0.05).

Total volatile content differed significantly with both diet and post-harvest treatment for perch under normal harvest conditions, that is, without heat shock (Figure 1). Frozen fillets showed the lowest total volatile content and relatively small differences between diets, due to limited volatile formation under −80 °C storage. Refrigerated storage increased total volatile content and increased the separation among diets, showing that diet-related differences became more apparent when oxidative, enzymatic, and microbial reactions were active. Baking produced the highest total volatile content, because thermal processing further increased volatile generation from precursors formed during refrigerated storage. Across the post-harvest treatments, fillets from fish fed the camu camu and wheat diets generally showed lower total volatile content, while fillets from fish fed the mixed starch and commercial diets showed higher total volatile content. Figure 1 therefore provides an overview of how post-harvest handling amplified diet-related differences in off-odor volatile formation.

To better understand how fillet composition affected volatile formation, antioxidant activity, vitamin E content, and lipid content of the muscle (fillet) were also measured for each diet and harvest condition.

Antioxidant capacity of yellow perch fillet was significantly influenced by both dietary treatment and use of heat shock (Table 2). Without heat shock, diet significantly affected fillet ABTS activity (F (3, 8) = 6.49, *p* = 0.0155); Tukey–Kramer HSD showed significant pairwise comparisons in camu camu vs. mixed starch (*p* = 0.0330) and wheat flour vs. mixed starch *p* = 0.0412. In this case, fish fed the camu camu and wheat flour diets exhibited the highest antioxidant activity (36.9% and 36.4%, respectively), followed by the commercial diet (28.5%), with the mixed starch diet lowest (26.5%). The higher antioxidant capacity in camu camu–fed fish reflects greater incorporation of dietary antioxidant compounds into muscle tissue; camu camu is a rich source of vitamin C (1.5–6 g/100 g dry weight) and polyphenolic compounds including ellagic acid, ellagitannins, and flavonoids [34,35,36], which are absorbed and distributed to muscle tissues. However, a number of phenolic compounds are water-soluble and heat-labile, which may not be effectively extracted using a lipophilic extract in the ABTS assay. Comparable antioxidant activity in wheat-fed fish may reflect phenolic compounds naturally present in wheat flour, including ferulic acid and other hydroxycinnamic acid derivatives [31,37]. The mixed starch diet is comprised of equal amounts of wheat, tapioca, and corn starch and produced the lowest antioxidant capacity; tapioca is a gluten-free amylopectin useful for pellet water-binding but not a significant source of bioactive compounds [38], and although corn has greater phenolic content than wheat [39], the overall bioactive contribution of the mixed starch formulation was lower than that of wheat- or camu camu–based diets.

Pre-harvest heat shock had a pronounced effect on muscle antioxidant capacity (Table 2). The pooled pre-harvest comparison (with vs. without heat shock) was significant (F (1, 22) = 54.56, *p* < 0.0001). Heat-shocked fish showed significantly elevated antioxidant activity (49.4%) compared with fish harvested without heat shock (32.1%), and the diet-dependent differences observed without heat shock were eliminated after applying heat shock, with all four groups ranging from 44.3–52.1% (F (3, 8) = 1.21, *p* = 0.3654). Acute thermal stress increases mitochondrial reactive oxygen species production and protein denaturation [40,41], prompting rapid upregulation of endogenous antioxidant defenses including heat shock proteins, superoxide dismutase, catalase, and glutathione peroxidase [42,43]. Acute heat shock has been shown to trigger tissue-specific increases in antioxidant enzymes in other fish species [41,44]. With heat shock, this stress response may be large enough to override the antioxidant differences established by diet, consistent with the disappearance of diet-dependent volatile differences with heat shock described in Section 3.2.

Alpha-tocopherol (vitamin E) content of the fillet was not significantly different among diets (F (3, 8) = 3.12, *p* = 0.0878, Table 3), although fish fed camu camu retained numerically higher levels (0.65 µg/g) than the mixed starch group (0.51 µg/g). Vitamin E is a chain-breaking antioxidant that protects polyunsaturated fatty acids from peroxidation in biological membranes [45]. Ascorbic acid interacts with oxidized alpha-tocopherol to get it back to its active form [46], so the higher dietary vitamin C supply in the camu camu diet may have protected vitamin E and prevented its depletion. This interaction of vitamins E and C has been demonstrated previously in yellow perch and Atlantic salmon [47,48], though the limited sample size (n = 3) constrains statistical power for this trend.

Muscle lipid content was significantly influenced by diet in fish harvested without heat shock (Table 4), with camu camu–fed fish showing the highest lipid content (3.42%) and mixed starch-fed fish the lowest (2.19%) (F (3, 8) = 4.54, *p* = 0.0372). Ascorbic acid in camu camu has been shown to increase activity of endogenous antioxidant enzymes while reducing lipid peroxidation in fish muscle and liver [49,50,51], which may have preserved a greater proportion of deposited lipid; vitamins C and E are known to interact to reduce lipid oxidation in yellow perch muscle specifically [48].

After heat shock, lipid content did not differ significantly among diets (3.21–3.80%) (F (3, 8) = 0.37, *p* = 0.7782). Comparing before and after heat shock, mixed starch and commercial diet fish showed an increase in lipid content. Elevated rearing temperature has been shown to reduce localized lipase activity in other species, limiting β-oxidation of circulating triglycerides and increasing intramuscular lipid accumulation in carp [52]. Wheat- and tapioca-based starches can also influence lipid metabolism independent of temperature, for example by upregulating lipogenic genes such as fatty acid synthetase, acetyl CoA-α [53] which may partly explain diet-related differences in lipid content alongside the antioxidant-related mechanisms already discussed [54]. (F (1, 22) = 6.24, *p* = 0.0204).

Fatty acid profiles of yellow perch fillet were more affected by the four diets than by the use of heat shock (Table 5). In terms of individual fatty acids, α-linolenic acid (C18:3n-3), EPA (C20:5n3) and DHA (C22:6n3) were higher in the mixed starch group. This phenomenon suggests the conversion of α-linolenic acid to longer-chain polyunsaturated fatty acids (PUFAs) through elongase and desaturase enzymes [55,56]. Although mixed starch showed higher long-chain fatty acid content, total PUFA content was higher in camu camu and wheat starch diets. To our knowledge, this is the first study to determine the effect of camu camu as a dietary source on the fatty acid profile in yellow perch muscle. Camu camu may protect against oxidation of long-chain PUFAs due to its higher antioxidant activity (Table 2 and Table 3), which may improve their retention in muscle. In a similar study on Nile tilapia (*Oreochromis niloticus*) juveniles, wheat starch upregulated the expression of lipogenic genes: peroxisome proliferator–activated receptor (PPARγ), diacylglycerol transferase (DGAT), and fatty acid synthetase (FAS)–driven lipogenesis, which can facilitate the esterification of free fatty acids into triglycerides by causing de novo fatty acid synthesis [57].

Heat shock had only a minor effect on the overall fatty acid composition (Table 5). Linoleic acid, EPA, and DHA were similar with and without heat shock. Heat shock– or temperature-driven effects mainly come from membrane remodeling effects by incorporating more PUFA in phospholipid composition [41,58]. As the duration of the heat shock was only 18 h (20 °C to 30 °C), it may not have been long enough to observe changes in muscle fatty acid composition.

Without heat shock, camu camu– and wheat-fed fish had generally higher antioxidant activity (Table 2), higher lipid content (Table 4), and lower volatile contents (Figure 1), while mixed starch– and commercial-fed fish showed lower antioxidant activity, lower lipid content, and higher volatile levels, especially during refrigerated storage and baking (Section 3.1.2 and Section 3.1.3).

#### 3.1.1. Frozen Fillets: Effect of Diet on the Volatile Profile Harvested Without Heat Shock Treatment

**Figure 2:**
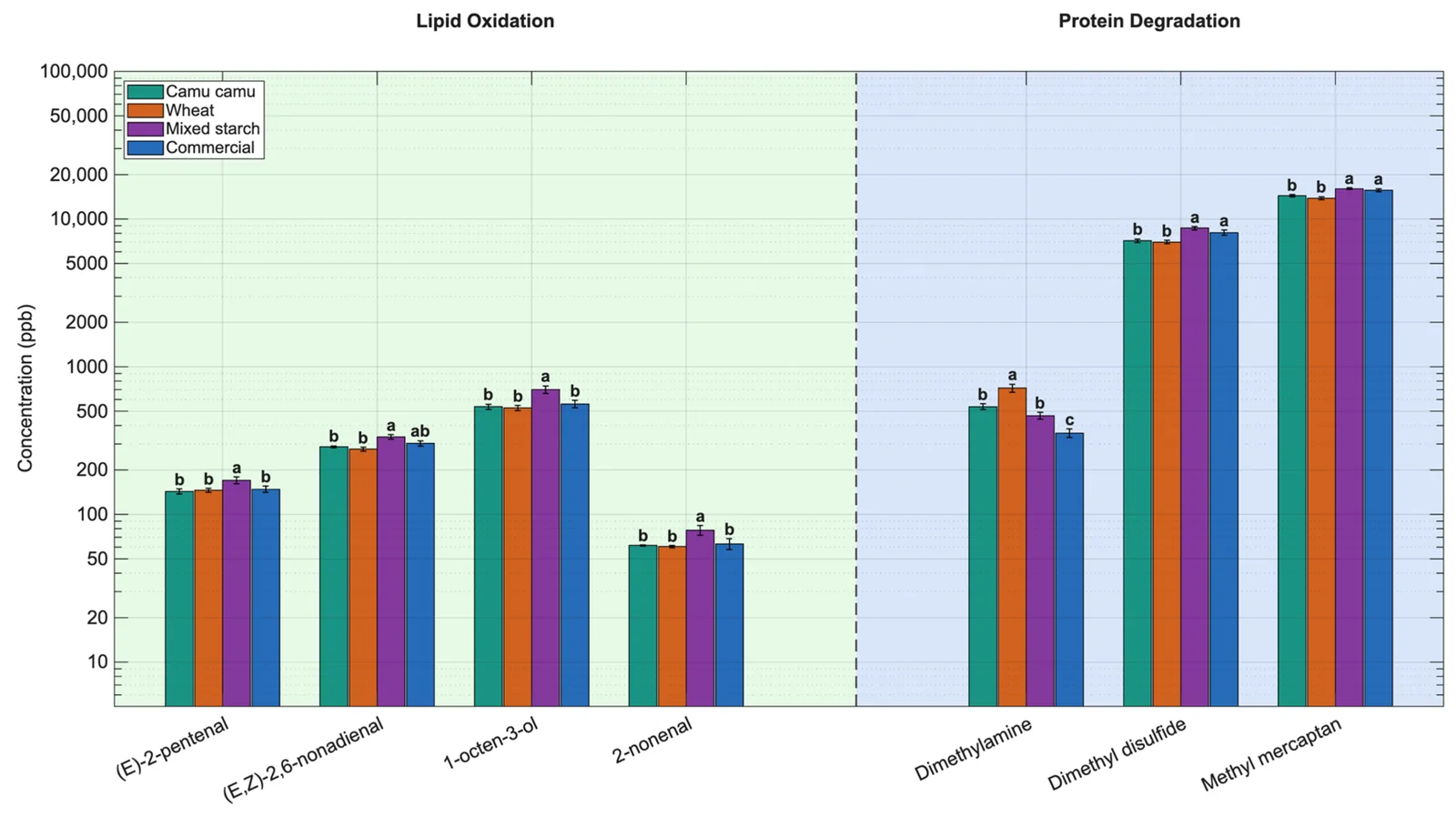
Effect of antioxidant-diet formulation on off-odor volatile compounds derived from lipid oxidation and protein degradation in frozen yellow perch fillets. Volatile concentration is plotted on a base-10 logarithmic (log10) scale. Values are means (n = 3 per diet). Different letters indicate significant differences in each volatile (*p* < 0.05).

Fillets from fish fed the different diets were frozen at −80 °C and thawed for 30 min to determine whether diet created differences in volatile concentrations. Very few diet-related differences were observed in frozen fillets across either the lipid oxidation markers (e.g., aldehydes, alcohols, ketones, hydrocarbons) or the protein degradation markers (e.g., sulfur compounds, amines) that make up the measured volatile profile [3] (Table A1, Figure 2). Both categories showed limited diet-related separation after frozen storage. Because the samples were immediately frozen and stored at −80 °C, microbial activity was halted [59,60], and enzymatic and oxidative reactions were substantially slowed [59,61]. Therefore, frozen fillets were used as a baseline to evaluate volatile levels before refrigerated storage and baking. The limited diet-related differences observed under frozen conditions indicate that antioxidant-diet effects were not strongly expressed when post-harvest volatile formation was minimized. This limited expression of diet-related volatile differences occurred despite statistically significant differences in fillet antioxidant activity and lipid content among diets (Table 2 and Table 4), indicating that composition differences alone were not sufficient to produce measurable volatile differences without processes such as refrigerated storage or baking, which increase volatiles through oxidative and enzymatic activity.

#### 3.1.2. Refrigerated Fillets: Effect of Diets on the Volatile Profile Harvested Without Heat Shock Treatment

**Figure 3:**
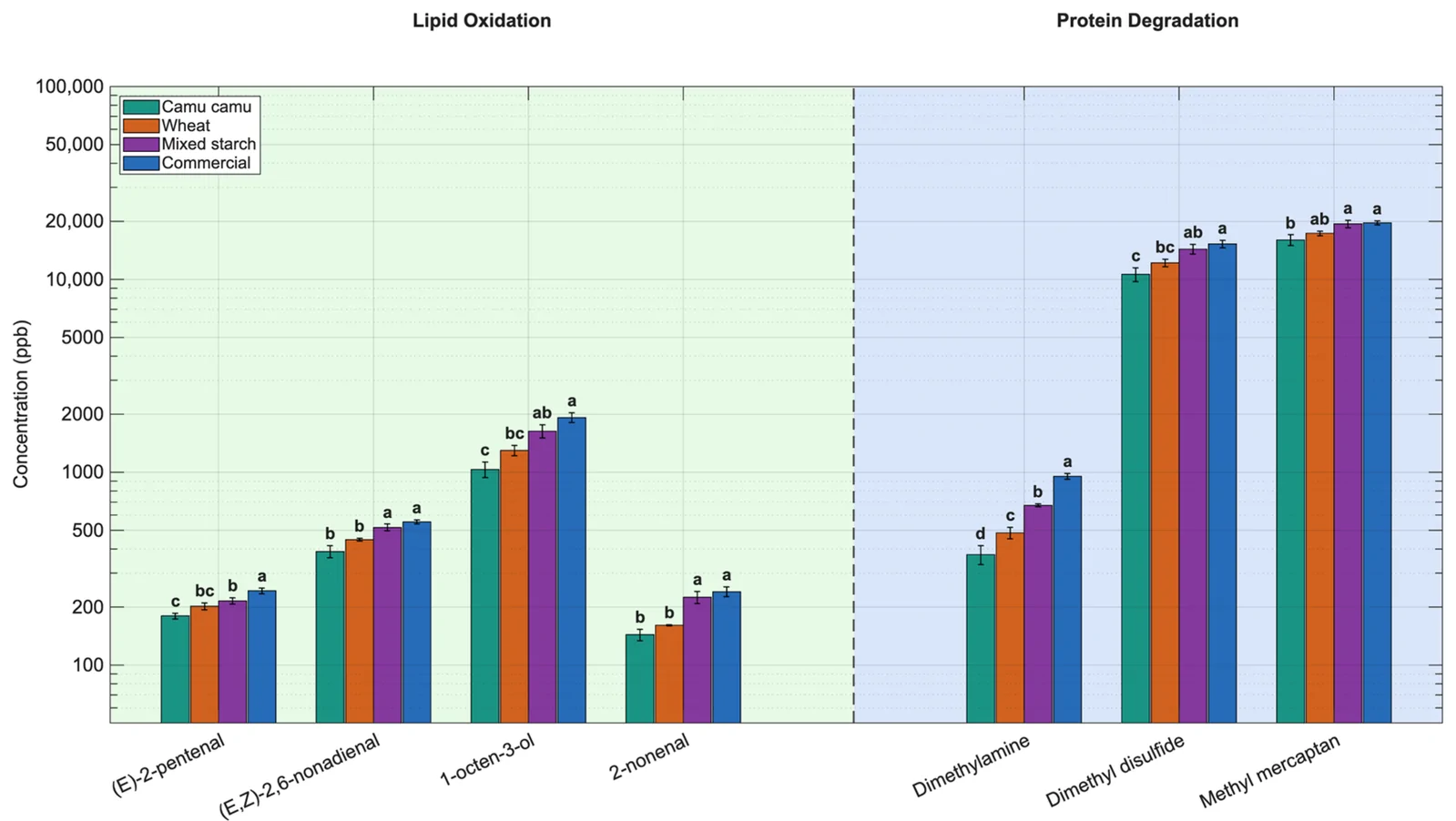
Effect of antioxidant-diet formulation on off-odor volatile compounds derived from lipid oxidation and protein degradation in refrigerated yellow perch fillet. Volatile concentration is plotted on a base-10 logarithmic (log10) scale. Values are means (n = 3 per diet). Different letters indicate significant differences in each volatile (*p* < 0.05).

Refrigerated storage at 4 °C for 3 days substantially increased volatile concentrations compared with frozen fillets and increased diet-related differences across the volatile profile (Table A2, Figure 3). Qualitatively, frozen fillets had little to no perceptible odor upon thawing, whereas refrigerated fillets had a noticeably fishy smell. This sensory contrast is consistent with the substantially higher instrumental volatile concentrations measured in refrigerated compared with frozen fillets (Table A1 and Table A2, Figure 2 and Figure 3), and suggests that perceptible odor differences may develop with storage even when diet-related instrumental differences remain limited immediately after freezing. This increase in volatile concentration occurred because refrigeration slows, but does not stop, oxidative, enzymatic, and microbial reactions [62,63]. This pattern is consistent with previous fish fillet storage studies, where refrigerated storage increased lipid oxidation and protein degradation volatiles over 15 days, and ice or refrigerated storage produced higher volatile concentrations than frozen storage because oxidative and degradation reactions continued during chilled storage [62,63,64]. Under refrigerated conditions, lipid oxidation proceeds through hydroperoxide formation and decomposition, producing aldehydes, alcohols, ketones, and hydrocarbons [63,64,65], while protein degradation and microbial metabolism generate sulfur-containing compounds and amines [5,64,66]. Proteolysis can also release free amino acids and pro-oxidants such as iron, further accelerating oxidative reactions [63,67]. Therefore, differences in tissue antioxidant activity and precursor availability became more apparent during refrigerated storage.

Diet significantly affected 22 lipid oxidation volatiles and 9 protein degradation volatiles in refrigerated fillets (Table A2, Figure 3). Lipid oxidation volatiles were generally lowest in fillets from fish fed the camu camu diet, followed by the wheat diet, and highest in fillets from fish fed the mixed starch and commercial diets (Table A2, Figure 3). This pattern was consistent with frozen fillets. This trend was observed across several major lipid-derived aldehydes, alcohols, and hydrocarbons, indicating that refrigerated storage promoted oxidation of lipid-derived precursors and increased separation among diet treatments. This pattern indicates that the camu camu and wheat diets limited lipid-derived off-odor formation during refrigerated storage, while the mixed starch and commercial diets showed greater susceptibility to volatile formation.

The lower lipid oxidation volatile concentrations in camu camu–fed fillets (Figure 3) are likely related to the antioxidant composition of camu camu powder and the higher antioxidant activity observed in these fillets without heat shock (Table 2). These compounds can inhibit lipid oxidation during refrigerated storage through radical scavenging and metal chelation [68,69], which reduces lipid hydroperoxide formation and decomposition into secondary volatiles such as aldehydes, alcohols, ketones, and hydrocarbons. Because vitamin C is water-soluble, it can act in the aqueous phase of muscle tissue, where it may intercept reactive oxygen species and reduce pro-oxidant metal activity before oxidation propagates into lipid substrates [26]. This explains why camu camu–fed fillets showed the lowest lipid oxidation volatile concentrations during refrigerated storage, when oxidative reactions were active, but thermal degradation of vitamin C had not yet occurred.

Wheat-fed fillets also showed low lipid oxidation volatile concentrations (Figure 3), but the likely mechanism differed from camu camu. Ferulic acid and related phenolic acids can donate hydrogen atoms or electrons to stabilize lipid radicals [70,71], while tocopherols act as chain-breaking antioxidants in lipid phases and membranes by interrupting lipid peroxyl radical propagation [72,73]. Therefore, wheat-fed fillets likely had lower lipid oxidation volatiles because wheat-derived antioxidants protected lipid substrates more directly within lipid-rich tissue regions.

The mixed starch diet produced higher lipid oxidation volatile concentrations than camu camu and wheat during refrigerated storage (Figure 3). This diet contained the same basal vitamin mix and stabilized vitamin C source as the other experimental diets, but it did not include an additional antioxidant-rich ingredient source such as camu camu powder or wheat flour. Therefore, its higher volatile concentrations should not be interpreted as a complete absence of antioxidants, but rather as insufficient antioxidant contribution beyond the basal formulation. The lower antioxidant activity associated with this diet without heat shock (Table 2) suggests that the tissue entered refrigerated storage with less protection against lipid oxidation, allowing greater hydroperoxide formation and subsequent production of lipid-derived off-odor volatiles.

The commercial diet also produced high lipid oxidation volatile concentrations during refrigerated storage (Figure 3), driven by both lower fillet antioxidant activity and higher lipid content, though for a different underlying reason than the mixed starch diet. The commercial diet had a higher crude fat content (15%) than the experimental diets (12%) and, although it contained added vitamin E, stable vitamin C, and carotenoid pigments, fish fed the commercial diet still had lower fillet antioxidant activity (Table 2) than fish fed the camu camu and wheat diets without heat shock. Therefore, the higher lipid oxidation volatiles in commercial-fed fillets likely reflect the diet’s higher measured lipid content (Table 4) combined with lower measured fillet antioxidant activity (Table 2), rather than a simple absence of antioxidants in the feed.

Protein degradation volatiles also showed pronounced diet effects under refrigerated storage (Table A2, Figure 3). These compounds were generally lowest in fillets from fish fed the camu camu and wheat diets, intermediate in fillets from fish fed the mixed starch diet, and highest in fillets from fish fed the commercial diet (Table A2, Figure 3). The increase in sulfur-containing and amine-related volatiles during refrigerated storage reflects activation of microbial and enzymatic degradation pathways. Methionine and cysteine degradation can generate methyl mercaptan, dimethyl disulfide, and related sulfur compounds [3,74,75], while trimethylamine can form through bacterial reduction of trimethylamine N-oxide during chilled storage [3,76]. This pattern indicates that diet-related differences during refrigerated storage were not limited to lipid oxidation but also extended to protein degradation pathways. Although antioxidants are primarily expected to limit lipid oxidation, lipid oxidation and protein degradation are closely linked during refrigerated storage. Lipid oxidation products can react with protein functional groups, while proteolysis can release free amino acids and pro-oxidant metals such as iron that further accelerate oxidation [65,77,78,79]. Therefore, diets that reduced lipid oxidation may also have indirectly reduced the formation of protein degradation-related off-odor volatiles by limiting oxidative damage and precursor formation during chilled storage.

#### 3.1.3. Baked Fillets: Effect of Diets on the Volatile Profile Harvested Without Heat Shock Treatment

**Figure 4:**
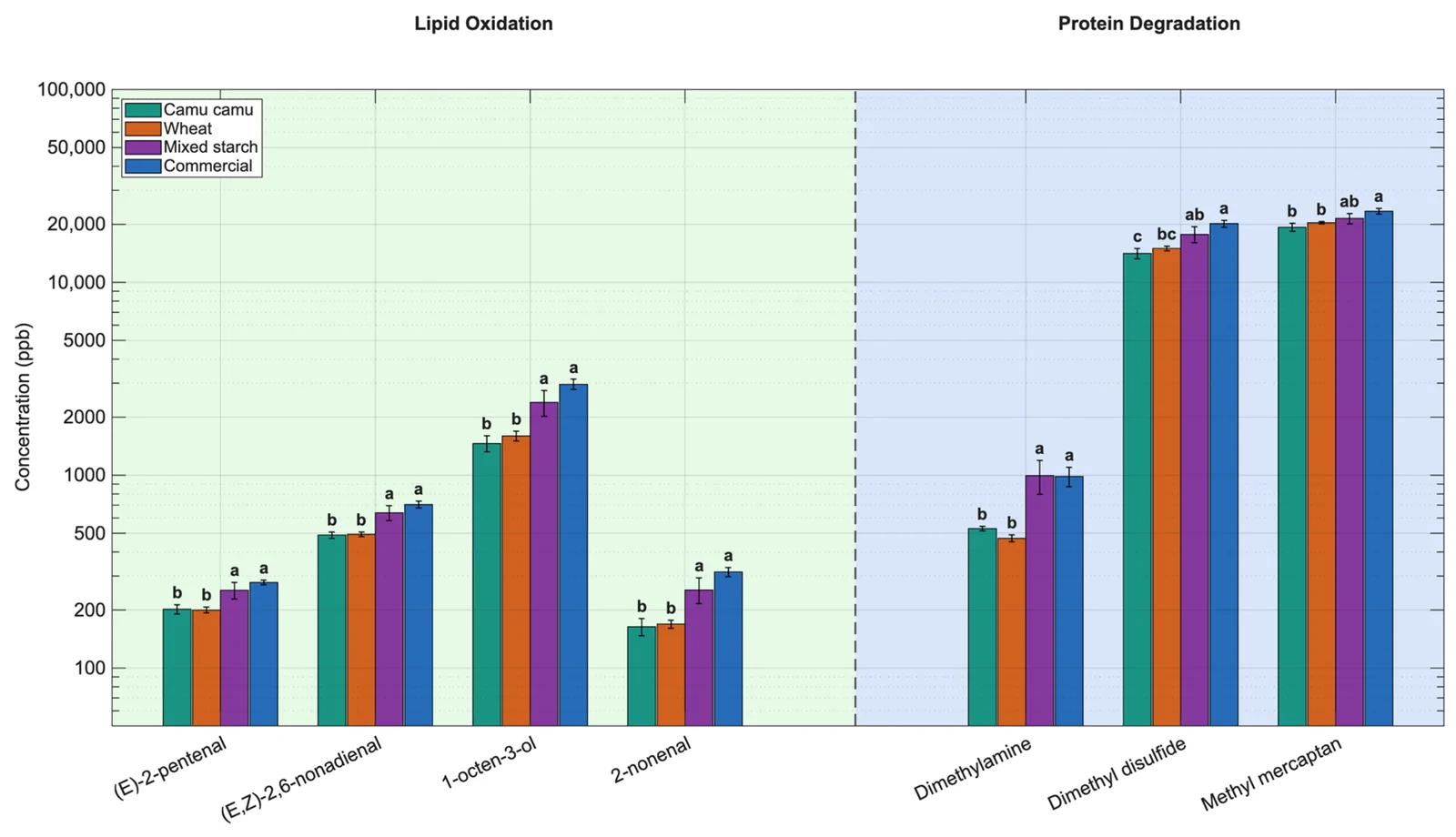
Effect of antioxidant-diet formulation on off-odor volatile compounds derived from lipid oxidation and protein degradation in baked yellow perch fillets. Volatile concentration is plotted on a base-10 logarithmic (log10) scale. Values are means (n = 3 per diet). Different letters indicate significant differences in each volatile (*p* < 0.05).

Baking at 200 °C for 15 min produced the highest volatile concentrations (Table A3, Figure 4). This increase occurred because heating rapidly converts lipid- and protein-derived precursors into volatile compounds [15,80]. Lipid hydroperoxides formed during refrigerated storage can decompose during baking to produce aldehydes, alcohols, ketones, hydrocarbons, and furans [15,81], while free amino acids and other protein degradation products can participate in Strecker degradation and Maillard reactions to form aldehydes, sulfur-containing compounds, nitrogen-containing compounds, pyrazines, and furan derivatives [15,81,82,83]. Therefore, the measured volatile profile of baked fillets reflected both precursor accumulation before cooking and thermal conversion of those precursors during baking.

Diet significantly affected 16 lipid oxidation volatiles and 8 protein degradation volatiles in baked fillets (Table A3, Figure 4). Volatile concentrations were generally lowest in fillets from fish fed the camu camu and wheat diets, intermediate in fillets from fish fed the mixed starch diet, and highest in fillets from fish fed the commercial diet. This pattern was consistent with the refrigerated fillets, indicating that diets that limited precursor formation during refrigerated storage also reduced volatile formation during baking. This pattern is consistent with the fillet antioxidant activity and lipid content differences measured among diets (Table 2 and Table 4), reinforcing that diets associated with higher antioxidant activity and lower lipid substrate availability without heat shock produced lower volatile concentrations across both refrigerated storage and baking.

Lipid oxidation volatiles followed the same general diet pattern across aldehydes, alcohols, ketones, and hydrocarbons, indicating that the diet effect was not limited to one lipid-derived compound class. These compounds are formed through related lipid oxidation pathways, beginning with hydroperoxide formation during storage followed by thermal decomposition during baking. Protein degradation volatiles showed a similar overall pattern, suggesting that diet also influenced the accumulation of reactive protein-derived precursors before baking.

The difference in concentration of Maillard- and Strecker-derived compounds after baking was not due to differences in total dietary protein content, because the diets were formulated with similar protein levels. Instead, diet influenced the amount of reactive precursors present in the fillet before cooking, including lipid hydroperoxides, carbonyl compounds, free amino acids, and other degradation products.

The advantage of camu camu observed during refrigerated storage was reduced after baking, likely because part of its protection was associated with heat-sensitive vitamin C [84,85]. However, the overall diet pattern remained the same after baking, with camu camu- and wheat-fed fillets maintaining lower volatile concentrations than mixed starch- and commercial-fed fillets. Wheat-fed fillets maintained low volatile concentrations after baking, likely because wheat-derived phenolic acids and tocopherols are more lipid-associated and may provide greater protection within lipid-rich regions during heating [86,87]. Mixed starch-fed fillets likely accumulated more precursors during refrigerated storage because the diet lacked an additional antioxidant-rich ingredient source beyond the basal vitamin mix, which includes vitamin C. Commercial-fed fillets likely had greater oxidizable lipid substrate because the commercial diet’s higher crude fat content and marine oil ingredients could generate more lipid oxidation precursors before and during baking.

### 3.2. Effect of Heat Shock and Diet on Lipid- and Protein-Derived Off-Odor Volatile Compounds

**Figure 5:**
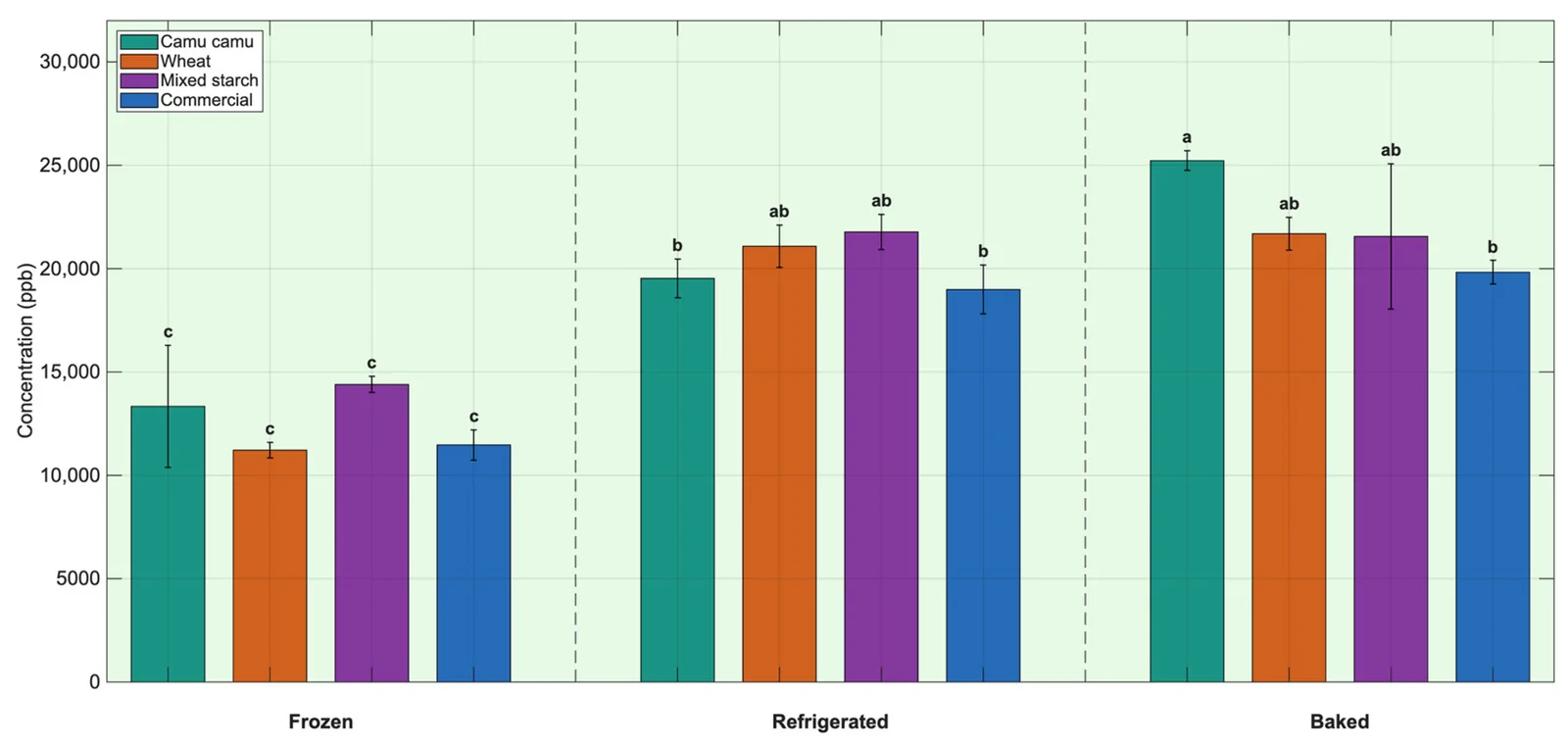
Effect of antioxidant-diet formulation on the total lipid-derived off-odor volatile content in frozen, refrigerated, and baked yellow perch fillets harvested with heat shock treatment. Values are means (n = 3 per diet). Different letters indicate significant differences (*p* < 0.05).

**Figure 6:**
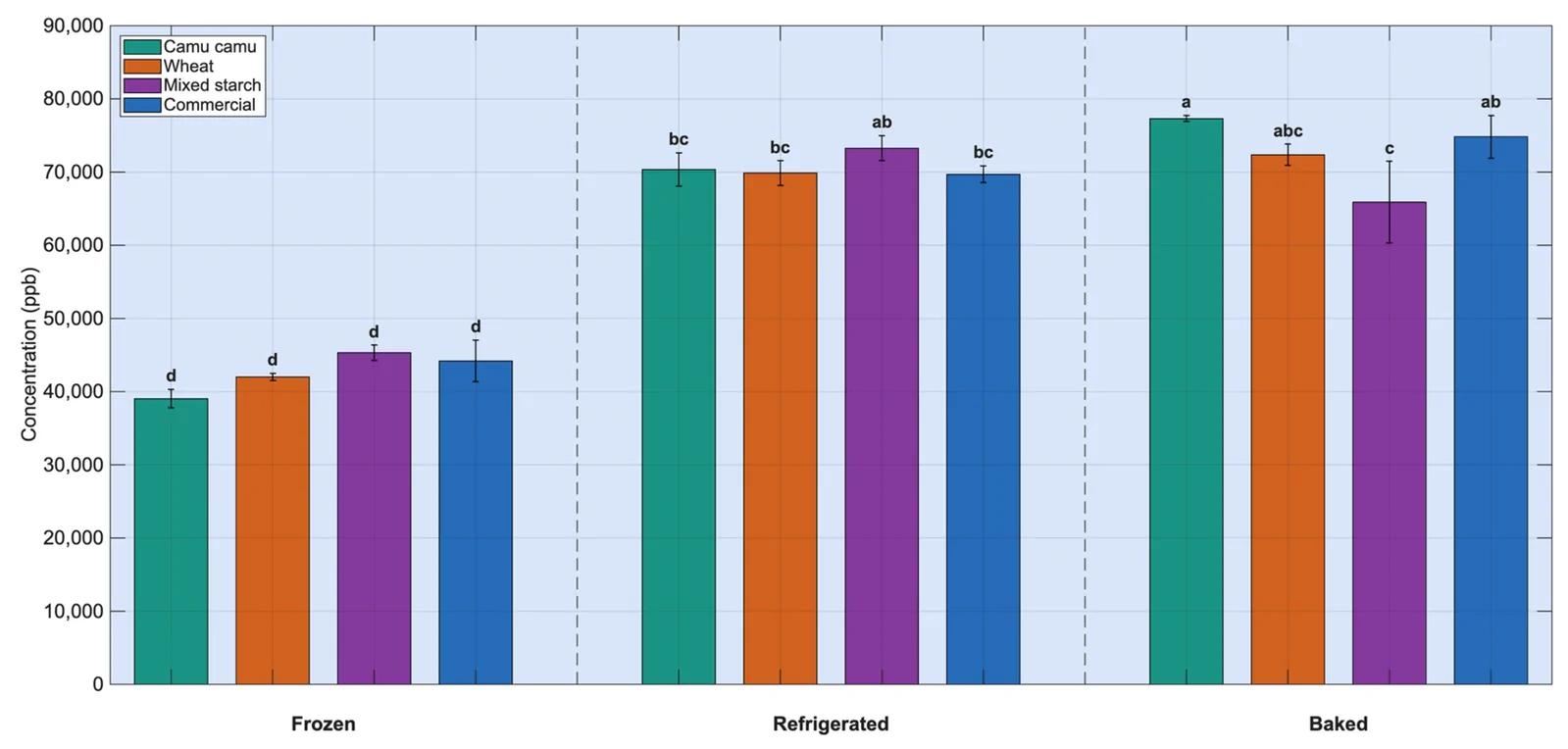
Effect of antioxidant-diet formulation on the total protein-derived off-odor volatile content in frozen, refrigerated, and baked yellow perch fillets harvested with heat shock treatment. Different letters indicate significant differences (*p* < 0.05).

A heat shock treatment was applied to evaluate tolerance to heat shock and its effect on lipid and protein oxidation in the final product. The potential of heat shock to promote depuration of environmental off-odor compounds such as geosmin and 2-methylisoborneol is addressed separately in Section 3.3. With heat shock, there were substantially reduced diet-driven differences in volatile profiles across all storage and baking conditions, compared to without heat shock (Figure 5 and Figure 6, Table A4, Table A5 and Table A6), indicating a fundamental shift in tissue chemistry due to the heat shock treatment prior to storage.

Heat shock significantly increased total volatile concentrations compared to the without heat shock harvest condition, indicating that acute thermal stress promoted overall off-odor volatile formation. Heat shock also eliminated diet-dependent differences across frozen, refrigerated, and baked fillets (Table A4, Table A5 and Table A6, Figure 5 and Figure 6). No major lipid oxidation or protein degradation markers were different across the four diets with heat shock, indicating that the diet-related separation observed without heat shock disappeared after acute thermal stress.

The disappearance of diet-dependent differences with heat shock is consistent with the antioxidant activity results. Without heat shock, tissue antioxidant activity differed among diets, with higher activity in fish fed the camu camu and wheat diets. With heat shock, however, ABTS radical scavenging activity increased across all diet groups and showed no difference, ranging from 44.3–52.1% (Table 2). This suggests that heat shock activated or altered the antioxidant response in all fish, causing fillets from the four diet groups to enter storage or cooking with a more similar antioxidant status. Because antioxidant status was not different across diets with heat shock, the influence of dietary antioxidant formulation on lipid oxidation and protein degradation volatile formation was no longer apparent. Therefore, under heat shock conditions, volatile formation was driven more by acute harvest stress and subsequent storage or cooking treatment than by diet.

### 3.3. Effect of Heat Shock and Diet on Environment-Derived Off-Odor Volatile Compounds

**Figure 7:**
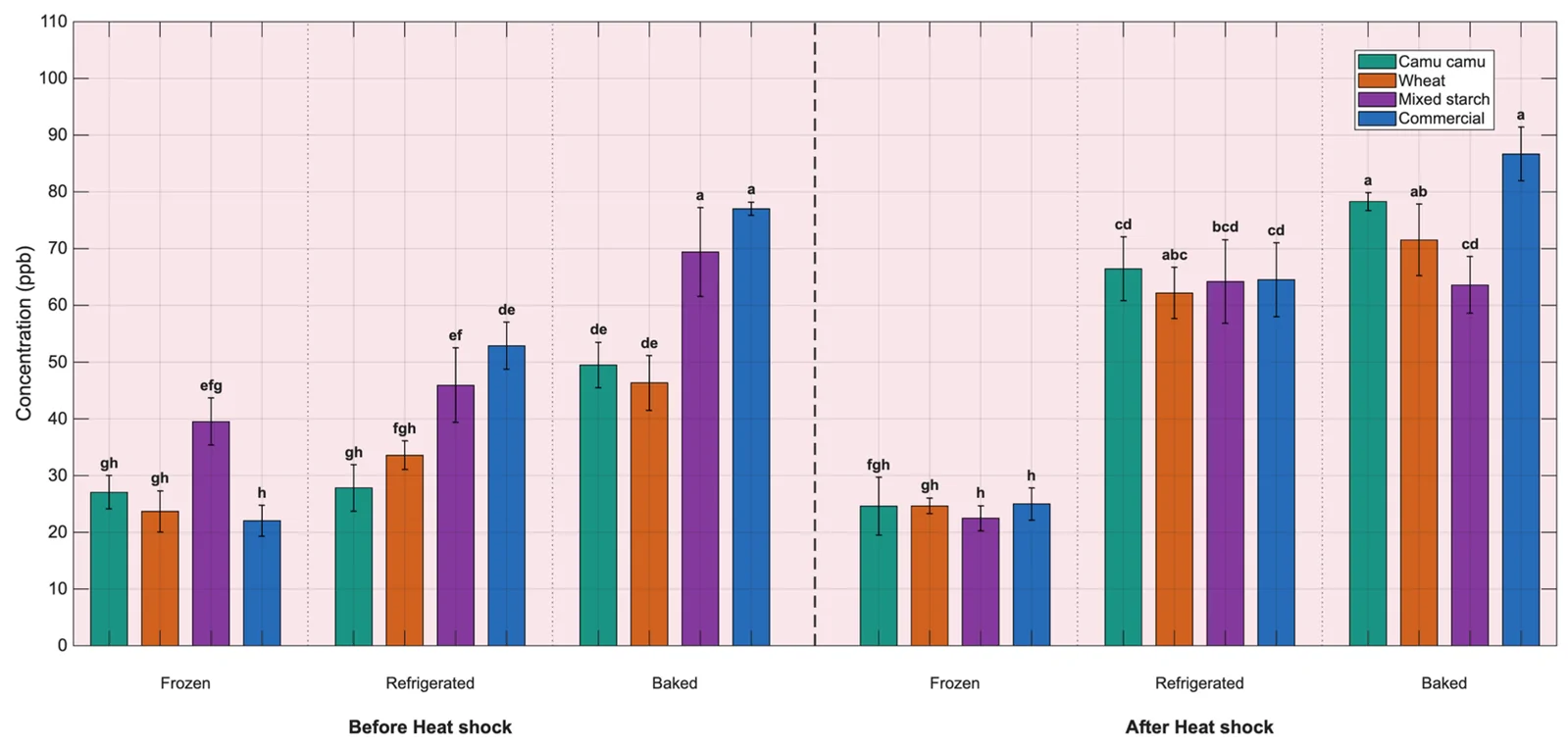
Effect of antioxidant-diet formulation on the total environmental off-odor volatile content in frozen, refrigerated, and baked yellow perch fillets harvested with and without heat shock treatment. Values are means (n = 3 per diet). Different letters indicate significant differences (*p* < 0.05).

Off-odor compounds that come from the environment, including geosmin and 2-methylisoborneol, did not show diet-dependent differences, regardless of whether the fillets were frozen, refrigerated, or baked, with or without heat shock (Figure 7, Table A1, Table A2, Table A3, Table A4, Table A5 and Table A6). This was expected because compounds such as geosmin and 2-methylisoborneol are primarily produced by microorganisms in the rearing environment, absorbed from water, and retained in fish tissue through lipid partitioning [10]. Therefore, unlike lipid oxidation and protein degradation volatiles, these environmental off-flavor compounds were not expected to respond to dietary antioxidant formulation. Because these compounds originate from microbial and environmental sources rather than lipid oxidation or protein degradation, they were not expected to track with the antioxidant activity, lipid content, or vitamin E differences measured among diets (Table 2, Table 3 and Table 4).

Heat shock was expected to reduce environmental off-flavor compounds by increasing diffusion and potential release of lipophilic compounds such as geosmin and 2-methylisoborneol into the surrounding water [10,88,89]. However, heat shock had no effect or slightly increased total environmental off-flavor volatile content in refrigerated and baked fillets (Figure 7, Table A4, Table A5 and Table A6). Total environmental off-flavor volatile content was calculated as the sum of geosmin, 2-methylisoborneol, α-terpinene, β-caryophyllene, 2-isopropyl-3-methoxypyrazine, and 2-methylnaphthalene. This indicates that the 18 h heat shock treatment at 30 °C was not effective as a depuration strategy and may have increased the measured concentration of environmental off-flavor compounds in the fillets. This increase may reflect redistribution or increased release of lipophilic compounds from tissue reservoirs. Because geosmin and 2-methylisoborneol strongly partition into lipid-rich tissues [64,88], effective removal requires sufficient time in clean, flowing water. The 18 h heat shock exposure was likely too short to achieve meaningful depuration.

Two primary factors explain this outcome. First, the duration of exposure was likely insufficient to achieve meaningful depuration. Previous studies have shown that effective removal of geosmin and 2-methylisoborneol from fish tissue typically requires several days to weeks in clean, flowing water, with even multi-day treatments often achieving only partial reduction [10,88,90]. Compared with these timescales, the 18 h heat shock exposure used in this study was too short to drive significant removal.

Second, the strong lipophilicity of geosmin and 2-methylisoborneol limits their rate of elimination from fish tissue. Both compounds preferentially partition into lipid stores, resulting in high retention within muscle tissue and slow diffusion back into the surrounding water [91,92]. Depuration therefore depends on gradual transfer from lipid to aqueous phases and subsequent elimination across gill and skin surfaces, a process that is inherently slow and further constrained by tissue lipid content [93].

Although elevated temperature during heat shock may increase membrane fluidity and diffusion rates, this effect alone was insufficient to overcome the thermodynamic and kinetic limitations governing these compounds. Temperature has limited impact on depuration efficiency compared with water exchange rate and exposure duration [89]. Therefore, similar or slightly higher concentrations observed in some heat-shocked samples likely reflect redistribution or increased release of lipophilic compounds from tissue reservoirs. Without sufficient time for diffusion-driven elimination, increased temperature may accelerate equilibration between tissue compartments and the headspace, resulting in similar or slightly higher measured concentrations.

### 3.4. Effect of Diets on Volatile Concentration and Antioxidant Activity of White Muscle, Liver, and Visceral Fat

Total volatile content was also measured directly in visceral fat and white muscle tissue to evaluate whether diet influenced volatile content formation in these tissues the way it did in the fillet. Diet did not significantly affect total volatile content in either tissue, with all four diets producing statistically similar volatile concentrations within each tissue type (Table 6).

To help explain the lack of diet-dependent volatile accumulation in visceral fat and white muscle, antioxidant activity was also measured in visceral fat, white muscle, and liver tissue to evaluate how dietary antioxidants were distributed across tissues with different metabolic roles (Table 7).

Antioxidant activity was highest in liver tissue, intermediate in white muscle, and lowest in visceral fat across all four diets. Within white muscle, camu camu- and wheat-fed fish showed the highest antioxidant activity, mixed starch-fed fish the lowest, and commercial-fed fish intermediate (F (3, 8) = 6.49, *p* = 0.0155; Table 7), matching the diet ranking observed in the fillet overall (Table 2).

Within liver, camu camu- and wheat-fed fish again showed the highest antioxidant activity, consistent with white muscle (F (3, 8) = 15.74, *p* = 0.0010). Commercial- and mixed starch-fed fish were both lowest in liver antioxidant activity and were not significantly different from each other (46.4 and 56.7, respectively), unlike in white muscle, where mixed starch alone was the distinct lowest group and commercial was intermediate. Most reactive oxygen species generation and detoxification occur in the liver, which increases its antioxidant activity relative to other tissues [94]. In Atlantic salmon, natural antioxidant supplementation significantly reduced malondialdehyde levels in both liver and muscle, indicating improved antioxidant protection across both tissues [95].

Visceral fat showed a different pattern from white muscle and liver: commercial-fed fish had the highest visceral fat antioxidant activity (17.5%) of the four diets, while mixed starch-fed fish remained the lowest (5.3%) (F (3, 8) = 6.45, *p* = 0.0157). Visceral fat is highly susceptible to oxidative stress and reactive oxygen species–driven lipid accumulation, which can deplete its endogenous antioxidant defenses and lower its baseline antioxidant activity relative to other tissues [96,97].

In visceral fat, similar to the fillet-level pattern described in Section 3.1, antioxidant activity and volatile content showed an inverse relationship: diets with higher antioxidant activity generally had lower volatile content, consistent with tissue antioxidant activity limiting volatile formation. The visceral fat relationship did not reach statistical significance (*p* = 0.0501), so it should be read as a numerical trend rather than a confirmed effect.

Overall, camu camu and wheat showed consistently high antioxidant activity across all three tissues, and mixed starch was consistently low in white muscle and visceral fat. Commercial was the outlier: at the fillet level (Section 3.1), commercial diet fish had higher measured lipid content (Table 4) and lower measured antioxidant activity (Table 2) than camu camu and wheat, but in visceral fat specifically, commercial diet fish showed the highest, not the lowest, antioxidant activity of the four diets.

### 3.5. Effect of Rearing Water System on Volatile Profile, Antioxidant Activity, Lipid Content and Fatty Acid Profile in Yellow Perch

**Figure 8:**
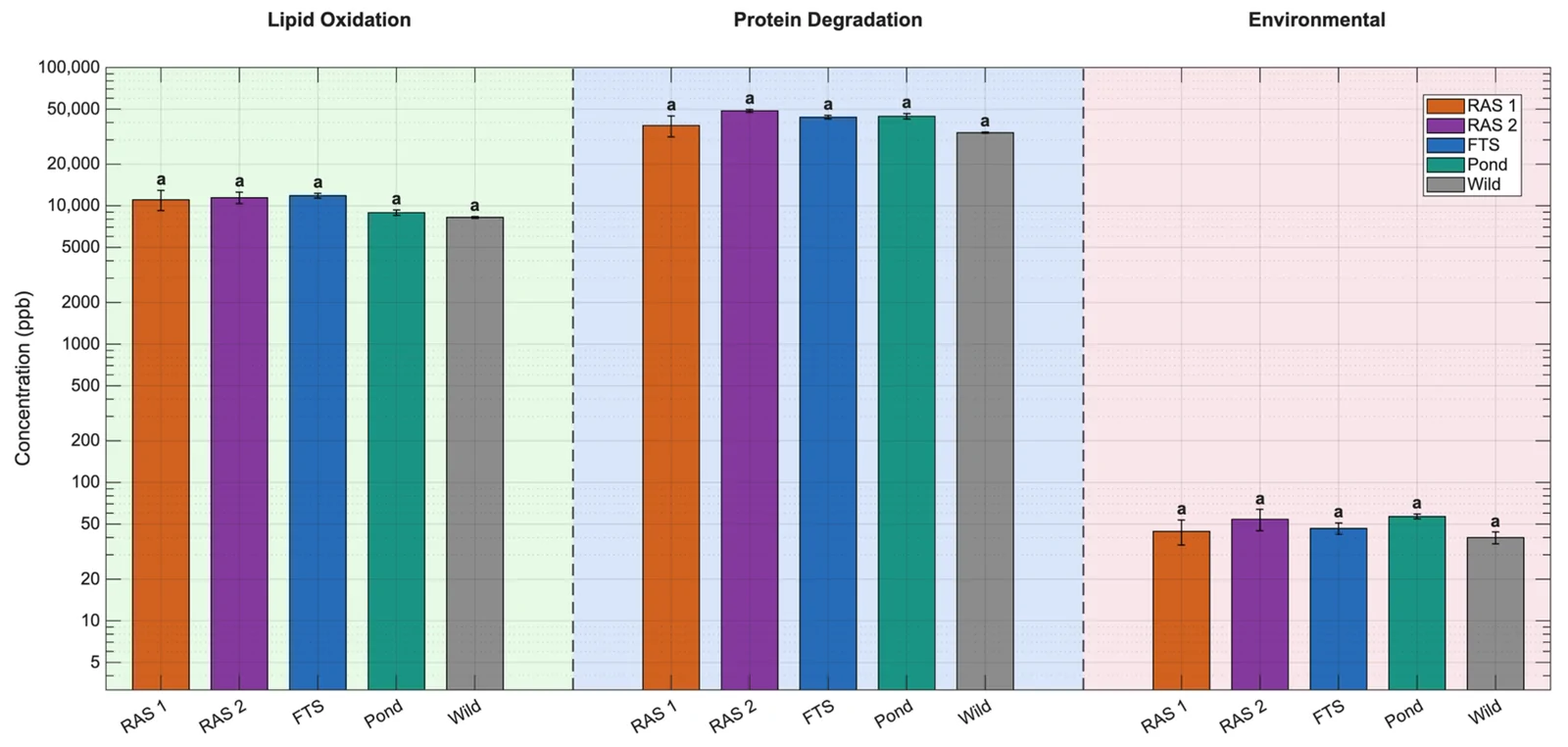
Effect of different rearing water systems on the total lipid, protein, and environmentally derived off-odor volatile content in yellow perch fillets. Volatile concentration is plotted on a base-10 logarithmic (log10) scale. Values are means (n = 3 per treatment). Different letters indicate significant differences within a volatile category (*p* < 0.05).

The effect of rearing water system on volatile formation in yellow perch fillets was evaluated by comparing fish raised in a flow-through system (FTS), recirculating aquaculture system (RAS 1, RAS 2), pond, and wild sources (Figure 8). Wild-caught fish were included as an additional comparison group representing an unmanaged, non-farmed condition. Overall, the rearing water system did not significantly affect the volatile compounds measured (lipid oxidation volatiles *p* = 0.1015, protein degradation volatiles *p* = 0.0532, and environmental contaminant volatiles *p* = 0.3841) (Figure 8). This suggests that the tested aquaculture systems produced volatile profiles comparable to wild fish. Environmental off-flavor compounds such as geosmin and 2-methylisoborneol can accumulate in recirculating aquaculture systems when microbial production and water quality conditions favor their formation [10,88,89]. However, in the present comparison, total environmental contaminant volatile content did not differ significantly among RAS, flow-through, pond, and wild fish, suggesting that a well-managed aquaculture system may limit accumulation of these compounds and produce yellow perch with environmental off-flavor levels comparable to wild fish. This is consistent with the depuration kinetics discussed in Section 3.3, where elimination of these lipophilic compounds was shown to depend more on water exchange rate than temperature. The continuous-flow systems evaluated here (Section 2.1.1) are therefore expected to limit their accumulation relative to more static systems. The tested aquaculture systems produced volatile profiles similar to wild-caught fish in this comparison, suggesting that well-managed rearing conditions are not inherently associated with elevated off-odor volatile content. However, because wild and farmed fish also differed in age, diet history, and genetic background, this should be interpreted as a source comparison rather than a controlled test of rearing system effects. Instead, off-odor formation in fish is likely influenced by multiple interacting rearing and postharvest factors, including diet, water quality, microbial activity, harvest handling, fish age, and storage conditions [64].

Muscle antioxidant capacity varied slightly among the five rearing environments evaluated for volatile formation (Table 8). Wild-caught fish showed the highest ABTS radical scavenging activity and was statistically the same as both recirculating aquaculture systems (RAS 1 and RAS 2) (F (4, 10) = 24.10, *p* ≤ 0.0001). Pond and flow-through system fish showed significantly lower antioxidant capacity than wild-caught and RAS-reared fish (*p* < 0.05). A previous comparison of traditional pond-based systems with RAS in Nile tilapia similarly found that antioxidant capacity was generally lower in the pond system [98]. The low antioxidant capacity of pond-reared fish in our study may reflect the more variable environmental conditions (temperature fluctuations, dissolved oxygen, pollutants) typical of outdoor pond systems compared with controlled indoor RAS facilities [99]. Such variability could produce chronic low-level oxidative stress that partially depletes antioxidant reserves without triggering the acute upregulation seen during heat shock [100]. The comparable antioxidant capacity of RAS-reared and wild-caught fish indicates that these controlled environments can produce fish with oxidative stability similar to wild populations, consistent with the volatile results above showing no significant difference in off-odor compounds among rearing systems. The significantly lower antioxidant capacity in flow-through system fish likely reflects an age effect rather than the rearing system itself: the flow-through fish sampled for this comparison were juveniles (4 months old), whereas the other groups were mature adults (2–5 years old), and antioxidant activity is known to increase as fish mature [101].

Muscle lipid content showed few differences among rearing environments (F (4, 10) = 4.73, *p* = 0.0211). Table 8). Wild-caught fish and those reared in the flow-through system showed the highest lipid content (2.88% and 2.86%, respectively); pond (2.43%) and RAS 1 (2.27%) fish were intermediate, and RAS 2 fish showed the lowest lipid content (1.62%), likely reflecting differences in diet formulation or feeding rate specific to that facility [5,102]. In a separate comparison of yellow perch reared in aquaponics, wild-caught, and traditional (non-recirculating) farm systems, lipid content was similarly comparable across all three rearing types (2.11–2.50%) [103]. As with antioxidant capacity, the comparable lipid content between wild-caught fish and most aquaculture systems indicates that controlled rearing environments can replicate the compositional characteristics of wild fish, and there are few significant differences in volatile concentrations, antioxidant, and lipid composition among rearing systems.

Polyunsaturated fatty acids, particularly docosahexaenoic acid (DHA, C22:6n3), were present in higher quantities in the RAS 1 system, while all other systems had similar levels of DHA to the wild fish (Table 9). Previous studies have shown that fatty acid composition is largely determined by feed and can be deliberately improved in farmed fish, unlike wild fish whose profile varies with prey and environment [104,105].

In terms of individual fatty acid contents, farmed fish showed a higher content of several key fatty acids, most notably docosahexaenoic acid (DHA), while wild-caught fish were richer in certain monounsaturated and saturated fatty acids. The docosahexaenoic acid (DHA, C22:6n3) content of the RAS fish was more than twice that of the wild-caught fish and gave the RAS fish the highest total n-3 PUFA and total PUFA content as well. As DHA is preferentially retained in fish tissue when supplied in the diet [106], this pattern demonstrates that a well-formulated feed can enrich cultured yellow perch in the very long-chain n-3 fatty acid. Eicosapentaenoic acid (EPA, C20:5n3) reinforced this point, as the RAS fish did not differ significantly from the wild-caught fish, confirming that farming did not compromise EPA status. Both farmed groups, therefore, matched or surpassed the wild fish fatty acid profile, restating the finding that farmed fish fed a well-formulated diet can provide a fatty acid profile similar to that of wild fish.

Based on the fatty acid class, farmed fish, particularly those in RAS 1 and pond, showed higher total PUFA and n-3 content, and both farmed groups were higher in n-6, compared to wild-caught fish (Table 9). However, wild-caught fish were higher in MUFA content, particularly from C16:1n-7 (palmitoleic acid), which is related to seasonal variations and is found more in warmer conditions [107]. In this study, wild-caught fish were harvested between July and August, which can increase their C16:1n-7 content. Effects of dietary intake and the fish’s capacity for endogenous PUFA biosynthesis from precursors are also documented in farmed rearing systems [108]. The core mechanism to increase PUFA content in fish muscle is, first, by increasing the PUFA content of the diet; second, by incorporating C18 precursors and allowing the fish’s own enzymes to upregulate and convert them to higher PUFAs via elongase and desaturase enzymes [55,56]. The commercial diets used in the flow-through system contained corn oil in the feed formulation, which is high (52%) in linoleic acid (C18:2n6) [109]. This high linoleic acid content appeared to increase the sum of n6 fatty acids in farmed fish, including flow-through systems, but was lower in wild-caught fish due to a lack of food sources high in linoleic acid. Wild-caught fish were higher in EPA content as well as the n3/n6 ratio due to access to algae, phytoplankton, and crustaceans, which are high in EPA content [110]. Apart from diet, water temperature is another important factor that can directly affect the fatty acid composition of fish muscle through homeoviscous adaptation [20,58,111]. Farmed conditions also allow precise control of photoperiod [112] and salinity [113] to upregulate endogenous PUFA biosynthesis, offering a higher level of nutritional control than wild-caught fish. The n-3/n-6 ratio of the RAS fish was similar to that of the wild-caught fish. Likewise, the PUFA/SFA ratio was similar among all five growing environments. Overall, combining all these factors, the results suggest that rearing environments such as RAS, flow-through, or pond can provide fish with a fatty acid profile that is comparable to wild-caught yellow perch.

### 3.6. Effect of Fish Weight on Volatile Formation in Yellow Perch

**Figure 9:**
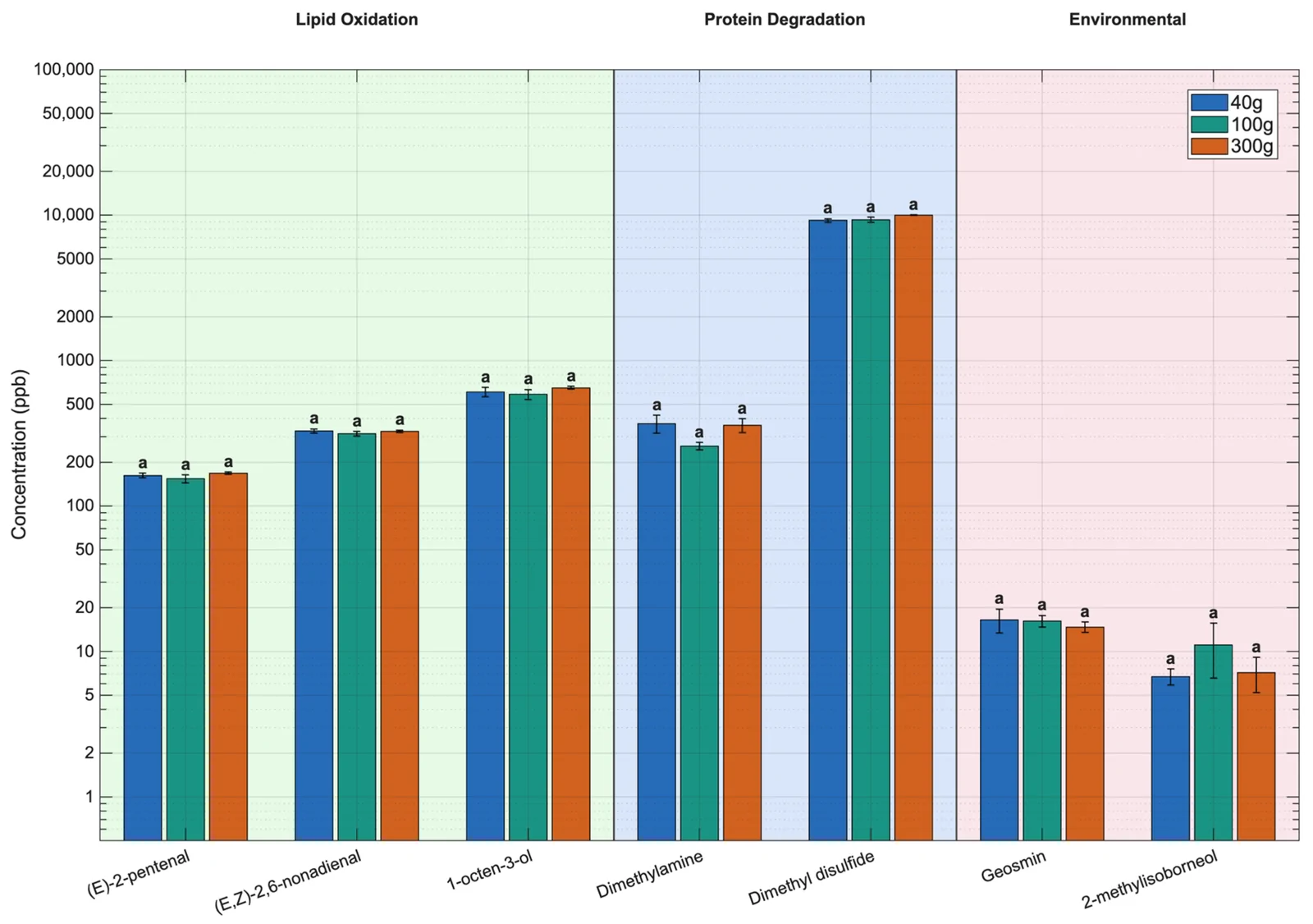
Effect of fish weight on the off-odor volatile compounds derived from lipid oxidation, protein degradation, and environmental contamination in yellow perch fillets. Volatile concentration is plotted on a base-10 logarithmic (log10) scale. Values are means (n = 3 per treatment). Different letters indicate significant differences in each volatile (*p* < 0.05).

To determine whether fish size at harvest influences volatile formation, yellow perch reared in the RAS system were sampled at three body weights (40 g, 100 g, and 300 g). Because all fish were adults and reared under the same diet and management conditions, results reflect the effect of size alone. Fish weight had no significant effect on the volatile profile in this comparison (*p* > 0.05) (Figure 9); however, with only three fish sampled per weight class, this study was not powered to detect small effects, and the result should be considered preliminary rather than conclusive evidence that volatile formation is unaffected by fish size. The results may indicate that the overall volatile profile is largely established by growth stage [5] and remains stable with fish size.

## 4. Conclusions

This study showed that off-odor volatile formation in yellow perch (*Perca flavescens*) fillets was influenced by diet, use of heat shock, and storage or cooking treatment. Storage or cooking treatment and heat shock significantly affected all volatile categories, while diet significantly affected lipid oxidation and protein degradation volatiles but not environmental contaminant volatiles. Without heat shock, the camu camu and wheat diets generally produced the lowest concentrations of lipid oxidation and protein degradation volatiles, indicating better control of off-odor formation during refrigerated storage and baking. The mixed starch and commercial diets produced higher volatile concentrations due to limited additional antioxidant contribution and higher lipid content in the case of the commercial diet. Higher antioxidant activity corresponded to lower volatile formation, without heat shock. Refrigerated storage and baking increased volatile formation, and diet-related differences became most apparent when oxidative, enzymatic, microbial, and thermal reactions were active.

Heat shock did not improve volatile quality. Instead, it increased volatile concentrations and reduced diet-dependent differences, likely because acute thermal stress altered tissue antioxidant status and reduced the relative advantage of antioxidant-rich diets. Environmental off-flavor compounds, including geosmin and 2-methylisoborneol, were not strongly diet-dependent because these compounds are primarily associated with environmental exposure and partitioning into fish tissue lipids. Short-duration heat shock was not effective for reducing these compounds, indicating that environmental off-flavor control requires water quality management and sufficient depuration rather than brief thermal treatment before harvest.

Fatty acid profile of yellow perch muscle was more affected by the dietary treatment than heat shock conditions because the short exposure time did not alter phospholipid composition. A higher content of long-chain fatty acids with limited antioxidant content may explain the higher volatile formation in mixed starch-fed yellow perch.

Rearing water systems and fish weight had limited effects on the overall volatile profile. The tested aquaculture systems did not produce significantly higher lipid oxidation, protein degradation, or environmental contaminant volatile contents than the wild reference, suggesting that well-managed aquaculture systems can produce yellow perch with volatile profiles comparable to wild fish. Fish weight similarly did not significantly affect volatile formation when fish were reared under the same growth stage, diet, and management conditions. Overall, these findings indicate that improving yellow perch flavor quality requires antioxidant-based diet strategies, low-stress harvest conditions, effective water quality management, and post-harvest handling that limits refrigerated and thermal off-odor formation.

## Figures and Tables

**Table 1:** Feed formulation (g/100 g) for three experimental diets used for a nine-week feeding study in yellow perch.

Ingredients	Wheat Flour	Camu Camu	Mixed Starch
Casein	32	32	32
Gelatin	8	8	8
Gluten	4	4	4
Menhaden meal	12	12	12
Fish hydrolysate	1.5	1.5	1.5
Squid meal: krill meal (1:1)	2	2	2
Sodium alginate	2	2	2
Carboxyl methylcellulose (CMC)	2	2	2
Stay C	0.2	0.2	0.2
Calcium phosphate dibasic	1	1	1
Vitamin mix (MP Bio)	3	3	3
Mineral mix (MP Bio)	2.2	2.2	2.2
Betaine hydrochloride	0.2	0.2	0.2
Tapioca starch	2	2	0
Choline chloride	1	1	1
Corn oil	5.5	5.5	5.5
Menhaden oil	5.5	5.5	5.5
Soy lecithin	1	1	1
Wheat flour	15		
Camu camu		15	
Mixed starch			17
Total	100	100	100

**Table 2:** Antioxidant activity (ABTS radical scavenging activity (RSA)) of yellow perch fillet fed four diets, with and without heat shock. Values are means (n = 3 per diet). Diets within each harvest condition were analyzed separately; different letters denote significant differences among diets (*p* < 0.05). The pooled pre-harvest condition rows show a significant overall difference between fish with and without heat shock (n = 12), with data pooled across diets (*p* < 0.05).

Harvest Condition	Diet	ABTS RSA (%)
Without heat shock	Camu camu	36.9 ^a^
Without heat shock	Wheat flour	36.4 ^a^
Without heat shock	Mixed starch	26.5 ^b^
Without heat shock	Commercial	28.5 ^ab^
With heat shock	Camu camu	49.7 ^a^
With heat shock	Wheat flour	51.5 ^a^
With heat shock	Mixed starch	44.3 ^a^
With heat shock	Commercial	52.1 ^a^
Pre-harvest condition (pooled)	With heat shock	49.4 ^a^
Pre-harvest condition (pooled)	Without heat shock	32.1 ^b^

**Table 3:** Vitamin E (alpha-tocopherol) content of yellow perch fillet from four dietary treatments without heat shock. Values are means (n = 3 per treatment). The same letter indicates no significant difference among treatments (*p* > 0.05).

Diet	Alpha-Tocopherol (µg/g)
Camu camu	0.65 ^a^
Wheat	0.56 ^a^
Mixed starch	0.51 ^a^
Commercial	0.55 ^a^

**Table 4:** Lipid content of yellow perch fillet fed four diets with and without heat shock. Values are means (n = 3 per diet). Diets within each harvest condition were analyzed separately; different letters denote significant differences among diets (*p* < 0.05); a thick black line marks the boundary between harvest conditions and the pooled comparison. The pooled pre-harvest condition rows show a significant overall difference between with and without heat shock (n = 12), with data pooled across diets (*p* < 0.05).

Harvest Condition	Diet	Lipid Content (%)
Without heat shock	Camu camu	3.42 ^a^
Without heat shock	Wheat flour	3.23 ^ab^
Without heat shock	Mixed starch	2.19 ^b^
Without heat shock	Commercial	2.85 ^ab^
With heat shock	Camu camu	3.21 ^a^
With heat shock	Wheat flour	3.69 ^a^
With heat shock	Mixed starch	3.80 ^a^
With heat shock	Commercial	3.63 ^a^
Pre-harvest condition (pooled)	Without heat shock	2.92 ^b^
Pre-harvest condition (pooled)	With heat shock	3.58 ^a^

**Table 5:** Fatty acid composition (% of fatty acid content) of yellow perch fillet fed four diets with and without heat shock. Values are means (n = 3 per treatment). Different letters indicate significant differences in each fatty acid (*p* < 0.05). Significance from two-way ANOVA (heat shock, diet, and their interaction) is indicated as: *** *p* < 0.001, ** *p* < 0.01, * *p* < 0.05, n/s = not significant. SFA, saturated fatty acids; MUFA, monounsaturated fatty acids; PUFA, polyunsaturated fatty acids; n3/n6, ratio of total n-3 to total n-6 fatty acids.

Fatty Acid Content (%)	Without Heat Shock				With Heat Shock				*p*-Values		
	Camu Camu	Wheat	Mixed Starch	Commercial	Camu Camu	Wheat	Mixed Starch	Commercial	Heat Shock	Diet	Interaction
C14:0	3.43 ^b^	3.46 ^b^	3.63 ^ab^	3.59 ^ab^	3.85 ^ab^	3.65 ^ab^	4.06 ^a^	3.58 ^ab^	**	*	n/s
C16:0	21.36 ^a^	21.94 ^a^	20.99 ^a^	20.81 ^a^	21.08 ^a^	21.01 ^a^	21.51 ^a^	21.23 ^a^	n/s	n/s	n/s
C16:1n7	8.33^c^	9.44 ^ab^	9.72 ^ab^	9.37 ^abc^	9.40 ^ab^	9.45 ^ab^	10.25 ^a^	8.93 ^bc^	n/s	***	*
C18:0	3.11 ^a^	3.12 ^a^	2.99 ^a^	2.91 ^a^	2.74 ^a^	2.76 ^a^	2.84 ^a^	2.96 ^a^	**	n/s	n/s
C18:1n9	24.93 ^a^	24.72 ^a^	25.25 ^a^	24.26 ^a^	23.51 ^a^	23.79 ^a^	24.53 ^a^	24.46 ^a^	*	n/s	n/s
C18:1n7	2.14 ^b^	2.19 ^b^	2.69 ^a^	2.13 ^b^	2.13 ^b^	2.16 ^b^	2.64 ^a^	2.12 ^b^	n/s	***	n/s
C18:2n6	19.36 ^ab^	17.98 ^b^	11.17^c^	18.56 ^ab^	20.17 ^a^	19.46 ^ab^	11.65^c^	18.86 ^ab^	**	***	n/s
C18:3n6	0.42 ^a^	0.45 ^a^	0.26 ^b^	0.47 ^a^	0.43 ^a^	0.42 ^a^	0.25 ^b^	0.42 ^a^	n/s	***	n/s
C18:3n3	0.95 ^b^	0.90 ^b^	1.31 ^a^	0.98 ^b^	1.01 ^b^	0.98 ^b^	1.38 ^a^	0.96 ^b^	*	***	n/s
C18:4n3	0.70 ^b^	0.69 ^b^	1.29 ^a^	0.74 ^b^	0.75 ^b^	0.74 ^b^	1.36 ^a^	0.47 ^b^	n/s	***	n/s
C20:0	0.15 ^ab^	0.13 ^b^	0.17 ^a^	0.13 ^b^	0.12 ^b^	0.12 ^b^	0.16 ^a^	0.13 ^b^	**	***	n/s
C20:1n9	0.73 ^b^	0.65 ^bc^	1.40 ^a^	0.66 ^bc^	0.59^c^	0.65 ^bc^	1.39 ^a^	0.64 ^bc^	*	***	*
C20:2n6	0.21 ^a^	0.19 ^abc^	0.17 ^bc^	0.20 ^ab^	0.18 ^abc^	0.18 ^abc^	0.16^c^	0.19 ^abc^	*	**	n/s
C20:3n6	0.39 ^a^	0.36 ^a^	0.20 ^b^	0.37 ^a^	0.35 ^a^	0.38 ^a^	0.20 ^b^	0.39 ^a^	n/s	***	n/s
C20:4n6	0.70 ^ab^	0.70 ^ab^	0.88 ^a^	0.72 ^ab^	0.67 ^b^	0.71 ^ab^	0.79 ^ab^	0.76 ^ab^	n/s	**	n/s
C20:4n3	0.39 ^bc^	0.34^c^	0.44 ^ab^	0.39 ^abc^	0.41 ^abc^	0.41 ^abc^	0.47 ^a^	0.41 ^abc^	*	**	n/s
C20:5n3	3.38 ^b^	3.34 ^b^	5.37 ^a^	3.47 ^b^	3.50 ^b^	3.50 ^b^	5.32 ^a^	3.56 ^b^	n/s	***	n/s
C22:4n6	0.16 ^a^	0.16 ^a^	0.17 ^a^	0.18 ^a^	0.17 ^a^	0.16 ^a^	0.16 ^a^	0.18 ^a^	n/s	n/s	n/s
C22:5n6	0.32 ^a^	0.31 ^ab^	0.25 ^ab^	0.31 ^ab^	0.30 ^ab^	0.32 ^a^	0.23 ^b^	0.33 ^a^	n/s	***	n/s
C22:5n3	1.09 ^a^	1.05 ^a^	1.18 ^a^	1.10 ^a^	1.14 ^a^	1.10 ^a^	1.14 ^a^	1.12 ^a^	n/s	*	n/s
C22:6n3	7.75^c^	7.87^c^	10.46 ^a^	8.32 ^bc^	7.49^c^	8.04^c^	9.53 ^ab^	8.32 ^bc^	n/s	***	n/s
Fatty acid class											
Ʃ SFA	28.05 ^a^	28.65 ^a^	27.79 ^a^	27.44 ^a^	27.79 ^a^	27.54 ^a^	28.57 ^a^	27.89 ^a^	n/s	n/s	n/s
Ʃ MUFA	36.13 ^b^	36.99 ^ab^	39.06 ^a^	36.43 ^b^	35.64 ^b^	36.06 ^b^	38.81 ^a^	36.16 ^b^	n/s	***	n/s
Ʃ PUFA	35.83 ^a^	34.36 ^ab^	33.15 ^b^	35.81 ^a^	36.57 ^a^	36.40 ^a^	32.63 ^b^	35.95 ^a^	n/s	***	n/s
Ʃ n3	14.27 ^b^	14.21 ^b^	20.05 ^a^	15.00 ^b^	14.30 ^b^	14.77 ^b^	19.20 ^a^	14.84 ^b^	n/s	***	n/s
Ʃ n6	21.56 ^ab^	20.15 ^b^	13.10^c^	20.80 ^ab^	22.27 ^a^	21.63 ^ab^	13.43^c^	21.12 ^ab^	*	***	n/s
n3/n6	0.66 ^b^	0.71 ^b^	1.53 ^a^	0.72 ^b^	0.64 ^b^	0.68 ^b^	1.43 ^a^	0.70 ^b^	*	***	n/s
PUFA /SFA	1.28 ^ab^	1.20 ^ab^	1.19 ^ab^	1.31 ^a^	1.32 ^a^	1.32 ^a^	1.14 ^b^	1.29 ^ab^	n/s	**	n/s

**Table 6:** Total volatile content in visceral fat and white muscle of yellow perch fed four diets. Values are means (n = 3 per diet). The same letter within each tissue indicates no significant difference among diets (*p* > 0.05).

Diet	Visceral Fat Mean (ppm)	White Muscle Mean (ppm)
Camu camu	143 ^a^	312 ^a^
Wheat	135 ^a^	343 ^a^
Mixed starch	144 ^a^	338 ^a^
Commercial	130 ^a^	254 ^a^

**Table 7:** Antioxidant activity (ABTS radical scavenging activity) in visceral fat, white muscle, and liver of yellow perch fed four diets. Values are means (n = 3 per diet). Different letters indicate significant differences among diets within each tissue column (*p* < 0.05); letters are not comparable across tissue columns.

Diet	Visceral Fat ABTS RSA (%)	White Muscle ABTS RSA (%)	Liver ABTS RSA (%)
Camu camu	10.9 ^ab^	36.9 ^a^	68.2 ^a^
Wheat	13.4 ^ab^	36.4 ^a^	65.8 ^ab^
Mixed starch	5.3 ^b^	26.5 ^b^	56.7 ^bc^
Commercial	17.5 ^a^	28.6 ^ab^	46.4 ^c^

**Table 8:** Muscle antioxidant activity (ABTS radical scavenging activity) of yellow perch from five different rearing environments. Values are means (n = 3 per treatment). Different letters indicate significant differences in each volatile (*p* < 0.05).

Growing Environment	ABTS RSA (%)	Lipid Content (%)
Wild caught	60.3 ^a^	2.88 ^a^
Pond	48.6 ^b^	2.43 ^ab^
Recirculating system 1 (RAS 1)	57.8 ^a^	2.27 ^ab^
Recirculating system 2 (RAS 2)	58.0 ^a^	1.62 ^b^
Flow-through system (FTS)	42.0 ^b^	2.85 ^a^

**Table 9:** Fatty acid content (mean ± SD, % of total fatty acids) of yellow perch from five rearing environments (recirculating aquaculture system, pond, and wild-caught). Values are means (n = 3 per treatment). Different letters indicate a significant difference in each fatty acid content (*p* < 0.05). Significance from one-way ANOVA is indicated as: *** *p* < 0.001, ** *p* < 0.01, * *p* < 0.05, n/s = not significant.

Fatty Acid Content (%)	Growing Environment					*p*-Values
	RAS 1	RAS 2	Flow-Through	Pond	Wild Caught	
C14:0	1.50 ^b^	3.12 ^a^	3.59 ^a^	3.22 ^a^	2.77 ^a^	**
C16:0	27.57 ^a^	21.74 ^b^	20.81 ^b^	20.48 ^b^	20.91 ^b^	***
C16:1n7	3.70 ^c^	8.23 ^b^	9.37 ^b^	11.32 ^b^	17.35 ^a^	***
C18:0	5.45 ^a^	3.40 ^b^	2.91 ^b^	2.33 ^b^	2.82 ^b^	***
C18:1n9	12.35 ^c^	22.13 ^ab^	24.26 ^a^	20.06 ^ab^	17.77 ^bc^	***
C18:1n7	1.71 ^c^	2.17 ^bc^	2.13 ^bc^	2.37 ^b^	5.20 ^a^	***
C18:2n6	10.88 ^b^	16.54 ^a^	18.56 ^a^	12.72 ^b^	3.79 ^c^	***
C18:3n6	0.39 ^a^	0.45 ^a^	0.47 ^a^	0.34 ^a^	0.36 ^a^	*
C18:3n3	0.71 ^c^	0.87 ^bc^	0.98 ^bc^	1.14 ^b^	2.38 ^a^	***
C18:4n3	0.27 ^b^	0.69 ^a^	0.74 ^a^	0.44 ^b^	0.85 ^a^	***
C20:0	0.08 ^b^	0.13 ^a^	0.13 ^a^	0.05 ^c^	0.11 ^a^	***
C20:1n9	0.40 ^b^	0.64 ^a^	0.66 ^a^	0.37 ^b^	0.44 ^b^	***
C20:2n6	0.20 ^a^	0.20 ^a^	0.20 ^a^	0.17 ^ab^	0.15 ^b^	*
C20:3n6	0.88 ^a^	0.44 ^b^	0.37 ^b^	0.40 ^b^	0.19 ^c^	***
C20:4n6	2.10 ^b^	1.01 ^c^	0.72 ^c^	2.28 ^b^	4.02 ^a^	***
C20:4n3	0.34 ^b^	0.42 ^ab^	0.39 ^ab^	0.37 ^ab^	0.47 ^a^	n/s
C20:5n3	4.82 ^ab^	4.34 ^bc^	3.47 ^c^	3.62 ^c^	5.73 ^a^	***
C22:4n6	0.53 ^ab^	0.31 ^bc^	0.18 ^c^	0.40 ^bc^	0.72 ^a^	**
C22:5n6	0.57 ^b^	0.41 ^b^	0.31 ^b^	0.57 ^b^	1.36 ^a^	***
C22:5n3	1.04 ^b^	1.24 ^b^	1.10 ^b^	1.47 ^ab^	1.93 ^a^	**
C22:6n3	24.53 ^a^	11.53 ^bc^	8.32 ^c^	15.87 ^b^	10.69 ^bc^	***
Fatty acid class						
Ʃ SFA	34.60 ^a^	28.39 ^b^	27.44 ^b^	26.08 ^b^	26.61 ^b^	***
Ʃ MUFA	18.15 ^b^	33.16 ^a^	36.43 ^a^	34.12 ^a^	40.76 ^a^	***
Ʃ PUFA	47.25 ^a^	38.45 ^b^	35.81 ^b^	39.80 ^ab^	32.63 ^b^	**
Ʃ n3	31.71 ^a^	19.09 ^b^	15.00 ^b^	22.90 ^b^	22.04 ^b^	***
Ʃ n6	15.54 ^b^	19.36 ^a^	20.80 ^a^	16.90 ^b^	10.59 ^c^	***
n3/n6	2.04 ^a^	0.99 ^bc^	0.72 ^c^	1.36 ^b^	2.09 ^a^	***
PUFA /SFA	1.37 ^ab^	1.36 ^ab^	1.31 ^ab^	1.52 ^a^	1.23 ^b^	n/s

## Data Availability

The data presented in the study are available in Appendix A. Further inquiries can be directed to the corresponding author.

## Appendix A

**Table A1:** Volatile profile of frozen yellow perch harvested without heat shock. Values are means (n = 3 per diet). Different letters indicate significant differences in each volatile (*p* < 0.05).

Volatile Compounds (ppb)	Camu Camu	Wheat	Mixed Starch	Commercial
(E)-2-pentenal	143 ^b^	146 ^b^	170 ^a^	148 ^b^
(E,Z)-2,6-nonadienal	287 ^b^	277 ^b^	335 ^a^	303 ^ab^
1-hexanol	14.6 ^ab^	11.5 ^ab^	19.9 ^a^	8.93 ^b^
1-octanol	7.21 ^a^	6.31 ^a^	10.7 ^a^	6.11 ^a^
1-octen-3-ol	537 ^b^	526 ^b^	701 ^a^	559 ^b^
1-octen-3-one	2.41 ^a^	1.29 ^a^	2.14 ^a^	1.48 ^a^
1-pentanol	20.3 ^b^	18.3 ^b^	33.5 ^a^	17.9 ^b^
1-penten-3-ol	70.2 ^b^	60.3 ^b^	132 ^a^	70.3 ^b^
2,3-butanediol	133 ^b^	114 ^b^	171 ^a^	130 ^b^
2,4-decadienal	2.67 ^ab^	1.44 ^b^	3.61 ^a^	1.79 ^ab^
2,4-heptadienal	358 ^ab^	237 ^b^	500 ^a^	261 ^b^
2-acetyl furan	4.32 ^ab^	4.02 ^ab^	5.81 ^a^	2.63 ^b^
2-butenal	11.3 ^b^	8.83 ^b^	16.9 ^a^	10.2 ^b^
2-decanone	1.19 ^ab^	1.34 ^a^	0.96 ^ab^	0.26 ^b^
2-decenal	2.81 ^a^	2.51 ^a^	3.45 ^a^	2.77 ^a^
2-ethyl-2,5,-dimethylpyrazine	2.67 ^ab^	3.05 ^ab^	4.65 ^a^	1.93 ^b^
2-ethylfuran	25.6 ^b^	26.5 ^b^	31.9 ^a^	28.1 ^ab^
2-heptanone	10.4 ^a^	11.3 ^a^	11.7 ^a^	10.1 ^a^
2-heptenal	26.4 ^ab^	22.8 ^b^	38.6 ^a^	19.1 ^b^
2-hexenal	25.3 ^b^	24.5 ^b^	31.6 ^a^	22.1 ^b^
2-isopropyl-3-methoxypyrazine	8.18 ^ab^	8.39 ^ab^	11.5 ^a^	6.62 ^b^
2-methyl naphthalene	15.8 ^ab^	20.2 ^ab^	23.0 ^a^	14.9 ^b^
2-methylisoborneol	13.4 ^b^	11.7 ^b^	23.1 ^a^	10.3 ^b^
2-nonanone	1.59 ^a^	2.33 ^a^	1.82 ^a^	1.26 ^a^
2-nonenal	61.7 ^b^	60.6 ^b^	78.2 ^a^	63.1 ^b^
2-octenal	4.73 ^ab^	4.59 ^ab^	7.73 ^a^	3.56 ^b^
2-octene	8.80 ^a^	10.5 ^a^	10.3 ^a^	7.56 ^a^
2-pentanone	2.09 ^a^	2.72 ^a^	2.35 ^a^	2.24 ^a^
2-penten-1-ol	51.8 ^b^	44.5 ^b^	97.2 ^a^	51.8 ^b^
2-pentene	30.1 ^b^	27.0 ^b^	49.5 ^a^	26.4 ^b^
2-undecanone	0.73 ^a^	1.06 ^a^	0.88 ^a^	1.19 ^a^
3-hexen-1-ol	2882 ^b^	2942 ^b^	3395 ^a^	3034 ^b^
3-hexenal	5.48 ^ab^	6.49 ^ab^	8.81 ^a^	4.63 ^b^
3-methyl-1-butanol	129 ^b^	95.8 ^b^	177 ^a^	108 ^b^
acetic acid	20.1 ^a^	16.7 ^a^	19.3 ^a^	17.2 ^a^
acetoin	6.27 ^a^	5.52 ^a^	6.32 ^a^	5.29 ^a^
acetone	36.4 ^a^	43.9 ^a^	46.0 ^a^	39.1 ^a^
alpha-terpinene	1.99 ^a^	1.58 ^a^	2.10 ^a^	1.45 ^a^
ammonia	787 ^c^	568 ^d^	1067 ^b^	1238 ^a^
beta-caryophyllene	0.54 ^a^	0.49 ^a^	1.13 ^a^	1.42 ^a^
carbon disulfide	1425 ^b^	1324 ^b^	1822 ^a^	1645 ^a^
decanal	1.88 ^a^	1.95 ^a^	2.43 ^a^	2.01 ^a^
dimethyl disulfide	7139 ^b^	6979 ^b^	8694 ^a^	8089 ^a^
dimethyl sulfide	4.77 ^bc^	2.60 ^c^	5.31 ^ab^	7.31 ^a^
dimethylamine	536 ^b^	718 ^a^	466 ^b^	355 ^c^
ethyl acetate	6.96 ^a^	6.13 ^a^	7.03 ^a^	5.88 ^a^
formaldehyde	1612 ^b^	1555 ^c^	1719 ^a^	1701 ^a^
geosmin	13.6 ^a^	12.0 ^a^	16.4 ^a^	11.8 ^a^
heptanal	6.42 ^a^	5.63 ^a^	7.20 ^a^	5.15 ^a^
heptane	388 ^b^	310 ^b^	546 ^a^	296 ^b^
hexanal	71.2 ^ab^	49.6 ^bc^	91.4 ^a^	34.1 ^c^
hexanoic acid	2.01 ^ab^	1.65 ^b^	2.59 ^a^	2.14 ^ab^
hydrogen sulfide	0.52 ^a^	0.40 ^a^	1.29 ^a^	0.61 ^a^
indole	0.52 ^b^	1.03 ^ab^	1.77 ^a^	1.35 ^ab^
isobutyl alcohol	13,306 ^b^	13,013 ^b^	15,167 ^a^	14,542 ^a^
methyl mercaptan	14,388 ^b^	13,841 ^b^	16,039 ^a^	15,676 ^a^
nonanal	18.2 ^ab^	17.4 ^b^	23.3 ^a^	18.5 ^ab^
oct-2-en-1-ol	464 ^b^	455 ^b^	606 ^a^	484 ^b^
octanal	6.10 ^b^	5.76 ^b^	8.85 ^a^	4.69 ^b^
octane	60.2 ^ab^	50.0 ^b^	79.5 ^a^	49.8 ^b^
pentanal	24.3 ^b^	21.8 ^b^	41.6 ^a^	23.2 ^b^
pentane	5961 ^b^	4734 ^c^	7149 ^a^	6100 ^b^
propanal	240 ^ab^	159 ^b^	336 ^a^	175 ^b^
trans-2-undecenal	8.47 ^b^	12.2 ^ab^	13.3 ^a^	11.3 ^ab^
trimethylamine	112 ^ab^	91.8 ^b^	137 ^a^	95.8 ^b^
trimethylpyrazine	21.1 ^b^	19.8 ^b^	27.2 ^a^	23.7 ^ab^

**Table A2:** Volatile profile of refrigerated yellow perch harvested without heat shock. Values are means (n = 3 per treatment). Different letters indicate significant differences in each volatile (*p* < 0.05).

Volatile Compounds (ppb)	Camu Camu	Wheat	Mixed Starch	Commercial
(E)-2-pentenal	180 ^c^	202 ^bc^	215 ^b^	243 ^a^
(E,Z)-2,6-nonadienal	388 ^b^	447 ^b^	517 ^a^	553 ^a^
1-hexanol	13.6 ^a^	12.9 ^a^	17.6 ^a^	12.6 ^a^
1-octanol	7.86 ^a^	9.41 ^a^	10.8 ^a^	10.4 ^a^
1-octen-3-ol	1034 ^c^	1297 ^bc^	1633 ^ab^	1920 ^a^
1-octen-3-one	1.03 ^a^	1.02 ^a^	1.11 ^a^	1.56 ^a^
1-pentanol	17.7 ^a^	19.3 ^a^	24.5 ^a^	25.3 ^a^
1-penten-3-ol	67.9 ^b^	94.0 ^ab^	117 ^a^	108 ^ab^
2,3-butanediol	121 ^a^	114 ^a^	133 ^a^	107 ^a^
2,4-decadienal	1.17 ^a^	1.26 ^a^	1.78 ^a^	1.98 ^a^
2,4-heptadienal	99.5 ^a^	93.7 ^a^	121 ^a^	89.0 ^a^
2-acetyl furan	2.62 ^b^	3.59 ^ab^	4.43 ^a^	4.72 ^a^
2-butenal	7.20 ^a^	9.27 ^a^	12.5 ^a^	9.83 ^a^
2-decanone	1.26 ^b^	1.94 ^ab^	1.32 ^ab^	2.46 ^a^
2-decenal	2.25 ^b^	3.12 ^ab^	3.92 ^a^	3.99 ^a^
2-ethyl-2,5,-dimethylpyrazine	3.53 ^bc^	3.32 ^c^	8.93 ^a^	6.94 ^ab^
2-ethylfuran	38.1 ^c^	43.7 ^bc^	49.6 ^ab^	56.5 ^a^
2-heptanone	8.14 ^a^	8.80 ^a^	9.46 ^a^	9.59 ^a^
2-heptenal	18.0 ^b^	21.1 ^b^	31.0 ^a^	30.3 ^a^
2-hexenal	41.1 ^c^	45.2 ^bc^	65.9 ^a^	61.3 ^ab^
2-isopropyl-3-methoxypyrazine	10.3 ^a^	11.1 ^a^	12.7 ^a^	19.8 ^a^
2-methyl naphthalene	17.0 ^a^	17.4 ^a^	25.1 ^a^	22.2 ^a^
2-methylisoborneol	6.80 ^a^	7.97 ^a^	9.40 ^a^	11.80 ^a^
2-nonanone	1.60 ^a^	1.68 ^a^	1.94 ^a^	1.77 ^a^
2-nonenal	144 ^b^	161 ^b^	225 ^a^	240 ^a^
2-octenal	2.30 ^b^	3.82 ^a^	3.35 ^ab^	2.97 ^ab^
2-octene	11.3 ^b^	15.9 ^a^	17.6 ^a^	19.1 ^a^
2-pentanone	1.93 ^a^	2.09 ^a^	2.71 ^a^	2.88 ^a^
2-penten-1-ol	50.1 ^b^	69.3 ^ab^	86.4 ^a^	79.7 ^ab^
2-pentene	26.1 ^a^	28.5 ^a^	36.3 ^a^	37.4 ^a^
2-undecanone	1.19 ^ab^	0.77 ^ab^	1.55 ^a^	0.46 ^b^
3-hexen-1-ol	3504 ^c^	3963 ^bc^	4243 ^b^	4800 ^a^
3-hexenal	3.24 ^a^	4.46 ^a^	4.28 ^a^	4.73 ^a^
3-methyl-1-butanol	64.4 ^a^	75.8 ^a^	89.7 ^a^	83.2 ^a^
acetic acid	17.1 ^a^	15.0 ^a^	14.7 ^a^	16.5 ^a^
acetoin	9.63 ^a^	9.13 ^a^	11.6 ^a^	9.05 ^a^
acetone	29.8 ^c^	40.7 ^b^	41.2 ^b^	53.1 ^a^
alpha-terpinene	3.13 ^a^	1.96 ^a^	1.75 ^a^	2.79 ^a^
ammonia	2204 ^a^	2333 ^a^	1683 ^a^	978 ^b^
beta-caryophyllene	0.52 ^a^	0.71 ^a^	0.96 ^a^	1.43 ^a^
carbon disulfide	2087 ^b^	2421 ^ab^	2809 ^a^	2874 ^a^
decanal	2.49 ^b^	3.18 ^ab^	3.56 ^ab^	4.81 ^a^
dimethyl disulfide	10,623 ^c^	12,189 ^bc^	14,348 ^ab^	15,271 ^a^
dimethyl sulfide	11.3 ^ab^	13.4 ^a^	8.10 ^bc^	4.55 ^c^
dimethylamine	374 ^d^	485 ^c^	673 ^b^	952 ^a^
ethyl acetate	10.7 ^a^	10.1 ^a^	12.9 ^a^	10.1 ^a^
formaldehyde	1557 ^b^	1612 ^b^	1764 ^a^	1763 ^a^
geosmin	21.0 ^b^	25.6 ^b^	36.5 ^a^	41.1 ^a^
heptanal	5.05 ^a^	6.07 ^a^	6.20 ^a^	6.71 ^a^
heptane	448 ^b^	501 ^ab^	710 ^ab^	717 ^a^
hexanal	48.4 ^a^	48.0 ^a^	66.6 ^a^	57.2 ^a^
hexanoic acid	1.90 ^a^	1.60 ^a^	3.16 ^a^	2.52 ^a^
hydrogen sulfide	1.11 ^a^	0.83 ^a^	0.27 ^a^	0.46 ^a^
indole	1.40 ^a^	2.28 ^a^	2.19 ^a^	2.50 ^a^
isobutyl alcohol	15,336 ^c^	17,050 ^bc^	19,378 ^ab^	20,027 ^a^
methyl mercaptan	15,983 ^b^	17,346 ^ab^	19,394 ^a^	19,650 ^a^
nonanal	22.9 ^b^	27.3 ^b^	38.8 ^a^	39.3 ^a^
oct-2-en-1-ol	893 ^c^	1120 ^bc^	1410 ^ab^	1658 ^a^
octanal	3.63 ^b^	6.17 ^a^	6.51 ^a^	5.68 ^a^
octane	59.5 ^c^	72.1 ^bc^	102 ^a^	89.9 ^ab^
pentanal	18.6 ^b^	19.0 ^ab^	26.0 ^a^	25.0 ^ab^
pentane	5449 ^a^	5439 ^a^	6421 ^a^	5090 ^a^
propanal	66.8 ^a^	63.0 ^a^	81.1 ^a^	59.8 ^a^
trans-2-undecenal	12.9 ^c^	14.6 ^bc^	24.3 ^a^	19.8 ^ab^
trimethylamine	45.0 ^a^	51.9 ^a^	54.9 ^a^	56.7 ^a^
trimethylpyrazine	33.1 ^b^	43.0 ^b^	54.7 ^a^	57.3 ^a^

**Table A3:** Volatile profile of baked yellow perch harvested without heat shock. Values are means (n = 3 per treatment). Different letters indicate significant differences in each volatile (*p* < 0.05).

Volatile Compounds (ppb)	Camu Camu	Wheat	Mixed Starch	Commercial
(E)-2-pentenal	202 ^b^	200 ^b^	253 ^a^	278 ^a^
(E,Z)-2,6-nonadienal	490 ^b^	494 ^b^	638 ^a^	705 ^a^
1-hexanol	7.08 ^a^	7.33 ^a^	7.97 ^a^	7.20 ^a^
1-octanol	5.19 ^a^	6.76 ^a^	8.10 ^a^	5.44 ^a^
1-octen-3-ol	1460 ^b^	1599 ^b^	2384 ^a^	2961 ^a^
1-octen-3-one	1.51 ^ab^	1.24 ^b^	2.74 ^a^	1.57 ^ab^
1-pentanol	31.0 ^a^	32.5 ^a^	41.6 ^a^	42.3 ^a^
1-penten-3-ol	91.4 ^a^	113 ^a^	135 ^a^	101 ^a^
2,3-butanediol	125 ^a^	128 ^a^	159 ^a^	151 ^a^
2,4-decadienal	2.12 ^a^	2.18 ^a^	2.44 ^a^	1.83 ^a^
2,4-heptadienal	119 ^a^	157 ^a^	111 ^a^	93.0 ^a^
2-acetyl furan	3.14 ^ab^	2.40 ^b^	4.63 ^a^	4.41 ^a^
2-butenal	4.57 ^a^	5.72 ^a^	5.34 ^a^	4.95 ^a^
2-decanone	2.23 ^ab^	0.69 ^b^	3.19 ^a^	3.60 ^a^
2-decenal	5.42 ^a^	6.03 ^a^	8.44 ^a^	7.17 ^a^
2-ethyl-2,5,-dimethylpyrazine	4.50 ^b^	6.17 ^ab^	9.37 ^a^	8.59 ^a^
2-ethylfuran	66.1 ^a^	62.3 ^a^	84.1 ^a^	78.2 ^a^
2-heptanone	11.6 ^a^	12.5 ^a^	13.6 ^a^	14.1 ^a^
2-heptenal	28.7 ^b^	32.9 ^ab^	41.8 ^a^	42.9 ^a^
2-hexenal	30.1 ^b^	29.7 ^b^	55.9 ^a^	47.8 ^ab^
2-isopropyl-3-methoxypyrazine	28.4 ^ab^	27.1 ^b^	38.2 ^ab^	39.7 ^a^
2-methyl naphthalene	19.7 ^b^	18.0 ^b^	32.5 ^a^	28.9 ^a^
2-methylisoborneol	13.0 ^a^	14.6 ^a^	13.9 ^a^	13.1 ^a^
2-nonanone	1.24 ^b^	1.25 ^b^	2.72 ^a^	2.18 ^ab^
2-nonenal	164 ^b^	169 ^b^	254 ^a^	315 ^a^
2-octenal	2.10 ^a^	2.88 ^a^	3.15 ^a^	2.95 ^a^
2-octene	17.9 ^b^	17.0 ^b^	21.6 ^ab^	28.3 ^a^
2-pentanone	5.79 ^a^	8.22 ^a^	8.92 ^a^	5.57 ^a^
2-penten-1-ol	67.4 ^a^	83.4 ^a^	99.5 ^a^	74.4 ^a^
2-pentene	45.8 ^a^	48.0 ^a^	61.4 ^a^	62.5 ^a^
2-undecanone	0.44 ^a^	1.00 ^a^	0.93 ^a^	0.40 ^a^
3-hexen-1-ol	3982 ^b^	4019 ^b^	5046 ^a^	5723 ^a^
3-hexenal	8.61 ^a^	9.61 ^a^	8.75 ^a^	7.78 ^a^
3-methyl-1-butanol	152 ^a^	197 ^a^	243 ^a^	174 ^a^
acetic acid	17.4 ^a^	18.8 ^a^	21.1 ^a^	22.3 ^a^
acetoin	5.80 ^a^	5.82 ^a^	7.36 ^a^	6.41 ^a^
acetone	66.8 ^a^	87.5 ^a^	104 ^a^	79.1 ^a^
alpha-terpinene	3.02 ^b^	3.41 ^ab^	4.49 ^ab^	4.85 ^a^
ammonia	1726 ^a^	1857 ^a^	1089 ^b^	1618 ^a^
beta-caryophyllene	1.07 ^ab^	0.18 ^b^	1.99 ^a^	1.67 ^ab^
carbon disulfide	3040 ^b^	3192 ^b^	3717 ^ab^	4214 ^a^
decanal	4.30 ^a^	3.48 ^a^	7.10 ^a^	5.59 ^a^
dimethyl disulfide	14,123 ^c^	14,999 ^bc^	17,720 ^ab^	20,177 ^a^
dimethyl sulfide	9.98 ^a^	8.46 ^a^	8.66 ^a^	7.24 ^a^
dimethylamine	529 ^b^	471 ^b^	995 ^a^	985 ^a^
ethyl acetate	6.44 ^a^	6.46 ^a^	8.18 ^a^	7.12 ^a^
formaldehyde	1723 ^b^	1889 ^ab^	1881 ^ab^	2056 ^a^
geosmin	36.5 ^b^	31.8 ^b^	55.5 ^a^	63.9 ^a^
heptanal	7.13 ^b^	6.70 ^b^	8.56 ^ab^	9.95 ^a^
heptane	3431 ^a^	4293 ^a^	6131 ^a^	3572 ^a^
hexanal	26.7 ^a^	35.1 ^a^	25.4 ^a^	23.0 ^a^
hexanoic acid	2.36 ^a^	1.75 ^a^	3.88 ^a^	2.67 ^a^
hydrogen sulfide	7.62 ^a^	18.04 ^a^	4.73 ^a^	3.86 ^a^
indole	0.54 ^b^	2.28 ^ab^	3.59 ^a^	2.70 ^ab^
isobutyl alcohol	19,306 ^b^	20,204 ^b^	22,530 ^ab^	24,776 ^a^
methyl mercaptan	19,296 ^b^	20,396 ^b^	21,455 ^ab^	23,382 ^a^
nonanal	36.4 ^b^	38.3 ^b^	51.2 ^ab^	56.2 ^a^
oct-2-en-1-ol	1261 ^b^	1382 ^b^	2058 ^a^	2555 ^a^
octanal	5.73 ^b^	6.56 ^b^	10.2 ^a^	7.86 ^ab^
octane	89.0 ^a^	101 ^a^	144 ^a^	132 ^a^
pentanal	104 ^a^	127 ^a^	159 ^a^	103 ^a^
pentane	7120 ^b^	7496 ^ab^	8432 ^a^	8603 ^a^
propanal	80.2 ^a^	106 ^a^	74.8 ^a^	62.7 ^a^
trans-2-undecenal	16.6 ^a^	18.1 ^a^	19.7 ^a^	24.4 ^a^
trimethylamine	88.3 ^a^	114 ^a^	124 ^a^	85.5 ^a^
trimethylpyrazine	52.8 ^b^	55.7 ^b^	73.6 ^a^	77.2 ^a^

**Table A4:** Volatile profile of frozen yellow perch harvested after head shock treatment. Values are means (n = 3 per treatment). Different letters indicate significant differences in each volatile (*p* < 0.05).

Volatile Compounds (ppb)	Camu Camu	Wheat	Mixed Starch	Commercial
(E)-2-pentenal	156 ^a^	163 ^a^	163 ^a^	170 ^a^
(E,Z)-2,6-nonadienal	303 ^a^	307 ^a^	316 ^a^	331 ^a^
1-hexanol	16.4 ^a^	12.5 ^a^	11.2 ^a^	10.0 ^a^
1-octanol	6.60 ^a^	6.33 ^a^	5.96 ^a^	6.33 ^a^
1-octen-3-ol	585 ^a^	597 ^a^	628 ^a^	652 ^a^
1-octen-3-one	2.66 ^a^	1.29 ^a^	1.59 ^a^	0.99 ^a^
1-pentanol	21.6 ^a^	15.6 ^a^	20.5 ^a^	19.1 ^a^
1-penten-3-ol	90.0 ^a^	67.7 ^a^	77.4 ^a^	77.8 ^a^
2,3-butanediol	170 ^a^	125 ^a^	194 ^a^	110 ^a^
2,4-decadienal	2.23 ^a^	1.98 ^a^	1.87 ^a^	2.10 ^a^
2,4-heptadienal	287 ^a^	197 ^a^	208 ^a^	201 ^a^
2-acetyl furan	6.24 ^a^	4.50 ^a^	4.69 ^a^	4.03 ^a^
2-butenal	12.5 ^a^	8.35 ^a^	10.5 ^a^	10.9 ^a^
2-decanone	1.04 ^a^	0.93 ^a^	1.14 ^a^	0.86 ^a^
2-decenal	4.19 ^a^	3.38 ^a^	3.13 ^a^	3.88 ^a^
2-ethyl-2,5,-dimethylpyrazine	3.45 ^a^	3.01 ^a^	2.65 ^a^	2.75 ^a^
2-ethylfuran	25.8 ^a^	29.1 ^a^	30.9 ^a^	29.6 ^a^
2-heptanone	9.13 ^a^	9.65 ^a^	9.87 ^a^	9.69 ^a^
2-heptenal	37.4 ^a^	22.7 ^a^	22.4 ^a^	20.6 ^a^
2-hexenal	24.3 ^a^	22.3 ^a^	23.0 ^a^	22.5 ^a^
2-isopropyl-3-methoxypyrazine	15.9 ^a^	8.39 ^a^	7.07 ^a^	8.18 ^a^
2-methyl naphthalene	20.1 ^a^	18.2 ^a^	16.5 ^a^	15.5 ^a^
2-methylisoborneol	11.0 ^a^	9.80 ^a^	7.92 ^a^	9.26 ^a^
2-nonanone	1.77 ^a^	1.00 ^ab^	1.21 ^ab^	0.48 ^b^
2-nonenal	62.9 ^a^	67.6 ^a^	75.1 ^a^	77.1 ^a^
2-octenal	7.51 ^a^	4.29 ^a^	5.00 ^a^	4.01 ^a^
2-octene	7.66 ^a^	8.49 ^a^	11.3 ^a^	10.1 ^a^
2-pentanone	3.21 ^a^	2.09 ^b^	2.01 ^b^	2.52 ^ab^
2-penten-1-ol	66.3 ^a^	49.9 ^a^	57.1 ^a^	57.4 ^a^
2-pentene	32.0 ^a^	23.0 ^a^	30.4 ^a^	28.2 ^a^
2-undecanone	0.76 ^a^	1.10 ^a^	1.33 ^a^	0.58 ^a^
3-hexen-1-ol	3088 ^a^	3243 ^a^	3207 ^a^	3431 ^a^
3-hexenal	6.57 ^a^	4.88 ^a^	4.86 ^a^	5.08 ^a^
3-methyl-1-butanol	144 ^a^	90.6 ^a^	94.1 ^a^	108 ^a^
acetic acid	24.3 ^a^	20.9 ^a^	25.1 ^a^	17.1 ^a^
acetoin	5.85 ^b^	6.23 ^b^	7.33 ^a^	5.74 ^b^
acetone	89.8 ^a^	41.3 ^ab^	33.7 ^b^	53.7 ^ab^
alpha-terpinene	1.93 ^a^	1.60 ^a^	2.50 ^a^	1.95 ^a^
ammonia	628 ^c^	1223 ^b^	1683 ^a^	1506 ^ab^
beta-caryophyllene	0.17 ^a^	1.09 ^a^	0.82 ^a^	0.91 ^a^
carbon disulfide	1516 ^a^	1580 ^a^	1761 ^a^	1715 ^a^
decanal	2.71 ^a^	2.26 ^a^	2.23 ^a^	1.82 ^a^
dimethyl disulfide	7161 ^a^	7819 ^a^	8521 ^a^	8433 ^a^
dimethyl sulfide	3.09 ^c^	5.78 ^b^	9.10 ^a^	6.81 ^ab^
dimethylamine	704 ^a^	502 ^b^	359 ^b^	425 ^b^
ethyl acetate	6.50 ^b^	6.92 ^b^	8.14 ^a^	6.38 ^b^
formaldehyde	1530 ^b^	1569 ^ab^	1670 ^a^	1623 ^ab^
geosmin	13.6 ^a^	14.8 ^a^	14.6 ^a^	15.7 ^a^
heptanal	6.68 ^a^	5.41 ^a^	5.10 ^a^	5.24 ^a^
heptane	463 ^a^	334 ^a^	524 ^a^	409 ^a^
hexanal	46.6 ^a^	46.8 ^a^	45.8 ^a^	31.8 ^a^
hexanoic acid	1.52 ^a^	1.65 ^a^	1.80 ^a^	1.53 ^a^
hydrogen sulfide	0.41 ^a^	0.30 ^a^	0.46 ^a^	1.10 ^a^
indole	1.08 ^a^	1.27 ^a^	1.19 ^a^	0.90 ^a^
isobutyl alcohol	13,192 ^b^	14,208 ^ab^	15,103 ^a^	14,798 ^ab^
methyl mercaptan	13,711 ^b^	14,733 ^ab^	15,777 ^a^	15,291 ^ab^
nonanal	17.8 ^a^	18.5 ^a^	19.1 ^a^	16.5 ^a^
oct-2-en-1-ol	506 ^a^	517 ^a^	543 ^a^	564 ^a^
octanal	9.58 ^a^	5.75 ^a^	6.03 ^a^	4.10 ^a^
octane	54.6 ^a^	51.3 ^a^	48.7 ^a^	47.2 ^a^
pentanal	36.2 ^a^	25.3 ^a^	36.0 ^a^	30.3 ^a^
pentane	7158 ^a^	5227 ^a^	8120 ^a^	5066 ^a^
propanal	193 ^a^	132 ^a^	140 ^a^	135 ^a^
trans-2-undecenal	7.12 ^a^	7.22 ^a^	6.36 ^a^	7.31 ^a^
trimethylamine	138 ^a^	74.8 ^b^	78.7 ^b^	90.0 ^ab^
trimethylpyrazine	22.0 ^a^	26.5 ^a^	27.4 ^a^	28.8 ^a^

**Table A5:** Volatile profile of refrigerated yellow perch harvested after head shock treatment. Values are means (n = 3 per treatment). Different letters indicate significant differences in each volatile (*p* < 0.05).

Volatile Compounds (ppb)	Camu Camu	Wheat	Mixed Starch	Commercial
(E)-2-pentenal	262 ^a^	251 ^a^	262 ^a^	245 ^a^
(E,Z)-2,6-nonadienal	636 ^a^	610 ^a^	655 ^a^	593 ^a^
1-hexanol	9.84 ^a^	14.4 ^a^	10.4 ^a^	11.9 ^a^
1-octanol	10.9 ^a^	15.8 ^a^	14.0 ^a^	11.3 ^a^
1-octen-3-ol	2414 ^a^	2358 ^a^	2577 ^a^	2106 ^a^
1-octen-3-one	1.14 ^a^	1.46 ^a^	1.53 ^a^	1.64 ^a^
1-pentanol	28.2 ^a^	38.6 ^a^	42.1 ^a^	33.3 ^a^
1-penten-3-ol	139 ^a^	249 ^a^	207 ^a^	196 ^a^
2,3-butanediol	159 ^a^	174 ^a^	165 ^a^	152 ^a^
2,4-decadienal	1.49 ^a^	1.79 ^a^	2.26 ^a^	1.68 ^a^
2,4-heptadienal	75.4 ^a^	124 ^a^	97.6 ^a^	113 ^a^
2-acetyl furan	6.48 ^a^	9.22 ^a^	11.09 ^a^	4.96 ^a^
2-butenal	11.8 ^a^	23.3 ^a^	16.5 ^a^	16.2 ^a^
2-decanone	1.62 ^a^	2.12 ^a^	1.51 ^a^	1.25 ^a^
2-decenal	3.26 ^a^	5.85 ^a^	5.66 ^a^	4.06 ^a^
2-ethyl-2,5,-dimethylpyrazine	10.0 ^a^	9.51 ^a^	11.5 ^a^	10.9 ^a^
2-ethylfuran	60.2 ^a^	59.6 ^a^	67.8 ^a^	62.3 ^a^
2-heptanone	13.0 ^a^	14.0 ^a^	12.8 ^a^	13.1 ^a^
2-heptenal	38.2 ^a^	45.1 ^a^	45.2 ^a^	32.2 ^a^
2-hexenal	28.0 ^a^	33.5 ^a^	31.7 ^a^	28.7 ^a^
2-isopropyl-3-methoxypyrazine	17.0 ^a^	29.1 ^a^	18.8 ^a^	18.9 ^a^
2-methyl naphthalene	29.5 ^a^	34.6 ^a^	30.4 ^a^	27.2 ^a^
2-methylisoborneol	10.2 ^a^	13.5 ^a^	10.7 ^a^	14.5 ^a^
2-nonanone	0.89 ^a^	1.51 ^a^	1.94 ^a^	1.18 ^a^
2-nonenal	237 ^a^	234 ^a^	253 ^a^	214 ^a^
2-octenal	4.54 ^a^	5.92 ^a^	5.06 ^a^	5.99 ^a^
2-octene	25.7 ^a^	24.2 ^ab^	24.9 ^a^	18.3 ^b^
2-pentanone	3.69 ^a^	3.81 ^a^	3.32 ^a^	3.31 ^a^
2-penten-1-ol	103 ^a^	183 ^a^	153 ^a^	145 ^a^
2-pentene	41.7 ^a^	57.0 ^a^	62.2 ^a^	49.2 ^a^
2-undecanone	1.32 ^a^	1.96 ^a^	1.31 ^a^	1.65 ^a^
3-hexen-1-ol	5256 ^a^	5112 ^a^	5333 ^a^	4789 ^a^
3-hexenal	4.35 ^a^	4.94 ^a^	4.09 ^a^	3.71 ^a^
3-methyl-1-butanol	122 ^a^	133 ^a^	103 ^a^	114 ^a^
acetic acid	20.5 ^ab^	23.7 ^a^	18.2 ^b^	21.0 ^ab^
acetoin	19.6 ^ab^	20.3 ^a^	19.5 ^ab^	14.6 ^b^
acetone	80.1 ^a^	64.2 ^a^	55.6 ^a^	66.9 ^a^
alpha-terpinene	3.36 ^a^	3.34 ^a^	3.37 ^a^	3.24 ^a^
ammonia	2803 ^a^	3070 ^a^	2846 ^a^	3583 ^a^
beta-caryophyllene	1.47 ^a^	0.95 ^a^	1.83 ^a^	1.50 ^a^
carbon disulfide	3458 ^a^	3346 ^a^	3478 ^a^	3440 ^a^
decanal	3.45 ^a^	1.89 ^b^	2.77 ^ab^	2.56 ^ab^
dimethyl disulfide	17,270 ^a^	17,078 ^a^	18,278 ^a^	16,714 ^a^
dimethyl sulfide	11.3 ^a^	13.9 ^a^	10.9 ^a^	15.4 ^a^
dimethylamine	918 ^a^	743 ^a^	890 ^a^	656 ^a^
ethyl acetate	21.8 ^ab^	22.6 ^a^	21.7 ^ab^	16.2 ^b^
formaldehyde	1907 ^a^	1868 ^a^	1933 ^a^	1873 ^a^
geosmin	56.2 ^a^	48.6 ^a^	53.5 ^a^	50.0 ^a^
heptanal	7.69 ^a^	7.56 ^a^	8.69 ^a^	8.79 ^a^
heptane	822 ^a^	1415 ^a^	1608 ^a^	853 ^a^
hexanal	20.7 ^a^	37.9 ^a^	25.5 ^a^	28.0 ^a^
hexanoic acid	2.40 ^a^	3.13 ^a^	2.65 ^a^	3.36 ^a^
hydrogen sulfide	1.49 ^ab^	1.20 ^b^	0.89 ^b^	4.70 ^a^
indole	2.01 ^a^	2.22 ^a^	3.52 ^a^	2.74 ^a^
isobutyl alcohol	22,173 ^a^	22,043 ^a^	23,137 ^a^	21,822 ^a^
methyl mercaptan	21,322 ^a^	21,194 ^a^	22,244 ^a^	21,122 ^a^
nonanal	48.4 ^a^	42.7 ^a^	48.3 ^a^	46.7 ^a^
oct-2-en-1-ol	2083 ^a^	2036 ^a^	2224 ^a^	1818 ^a^
octanal	6.48 ^a^	8.17 ^a^	7.55 ^a^	6.98 ^a^
octane	107 ^a^	101 ^a^	110 ^a^	95.0 ^a^
pentanal	28.8 ^a^	58.6 ^a^	56.7 ^a^	34.1 ^a^
pentane	6977 ^a^	7847 ^a^	7770 ^a^	7357 ^a^
propanal	50.6 ^a^	83.3 ^a^	65.6 ^a^	75.8 ^a^
trans-2-undecenal	23.5 ^a^	23.4 ^a^	21.5 ^a^	20.5 ^a^
trimethylamine	76.6 ^a^	75.0 ^a^	57.3 ^a^	74.1 ^a^
trimethylpyrazine	64.6 ^a^	60.9 ^a^	66.2 ^a^	64.4 ^a^

**Table A6:** Volatile profile of baked yellow perch harvested after head shock treatment. Values are means (n = 3 per treatment). Different letters indicate significant differences in each volatile (*p* < 0.05).

Volatile Compounds (ppb)	Camu Camu	Wheat	Mixed Starch	Commercial
(E)-2-pentenal	260 ^a^	261 ^a^	241 ^b^	250 ^ab^
(E,Z)-2,6-nonadienal	646 ^a^	637 ^ab^	550 ^b^	598 ^ab^
1-hexanol	9.47 ^a^	8.21 ^a^	9.21 ^a^	8.76 ^a^
1-octanol	6.67 ^a^	7.01 ^a^	6.69 ^a^	5.37 ^a^
1-octen-3-ol	2567 ^a^	2507 ^a^	1915 ^b^	2301 ^ab^
1-octen-3-one	1.38 ^a^	1.51 ^a^	1.57 ^a^	1.22 ^a^
1-pentanol	51.5 ^a^	36.2 ^ab^	39.4 ^ab^	32.8 ^b^
1-penten-3-ol	169 ^a^	125 ^a^	149 ^a^	108 ^a^
2,3-butanediol	130 ^a^	110 ^ab^	116 ^ab^	107 ^b^
2,4-decadienal	2.66 ^a^	2.66 ^a^	2.63 ^a^	2.66 ^a^
2,4-heptadienal	194 ^a^	136 ^a^	221 ^a^	223 ^a^
2-acetyl furan	5.17 ^a^	6.45 ^a^	4.70 ^a^	5.78 ^a^
2-butenal	8.07 ^a^	6.38 ^a^	6.73 ^a^	7.84 ^a^
2-decanone	2.98 ^a^	3.10 ^a^	0.58 ^a^	1.51 ^a^
2-decenal	8.26 ^a^	6.66 ^a^	5.56 ^a^	6.03 ^a^
2-ethyl-2,5,-dimethylpyrazine	10.9 ^a^	9.62 ^a^	6.20 ^a^	10.0 ^a^
2-ethylfuran	77.6 ^a^	69.8 ^ab^	64.3 ^b^	68.3 ^ab^
2-heptanone	14.8 ^a^	14.0 ^a^	13.0 ^a^	13.9 ^a^
2-heptenal	56.6 ^a^	41.3 ^ab^	39.7 ^b^	52.2 ^ab^
2-hexenal	44.6 ^a^	39.7 ^a^	34.8 ^a^	36.2 ^a^
2-isopropyl-3-methoxypyrazine	42.0 ^a^	33.2 ^ab^	24.5 ^b^	25.9 ^b^
2-methyl naphthalene	29.4 ^a^	33.3 ^a^	24.9 ^a^	30.1 ^a^
2-methylisoborneol	21.5 ^a^	18.4 ^a^	23.0 ^a^	31.4 ^a^
2-nonanone	1.71 ^a^	1.70 ^a^	2.47 ^a^	1.36 ^a^
2-nonenal	277 ^a^	267 ^ab^	209 ^b^	252 ^ab^
2-octenal	2.56 ^a^	3.39 ^a^	3.28 ^a^	3.09 ^a^
2-octene	26.5 ^a^	22.5 ^ab^	18.8 ^b^	22.8 ^ab^
2-pentanone	5.08 ^a^	6.17 ^a^	5.16 ^a^	3.55 ^a^
2-penten-1-ol	124 ^a^	92.2 ^a^	110 ^a^	79.8 ^a^
2-pentene	76.1 ^a^	53.5 ^ab^	58.2 ^ab^	48.4 ^b^
2-undecanone	1.23 ^a^	0.46 ^a^	1.46 ^a^	1.29 ^a^
3-hexen-1-ol	5096 ^ab^	5156 ^a^	4735 ^b^	5021 ^ab^
3-hexenal	6.67 ^b^	7.14 ^ab^	9.57 ^a^	7.16 ^ab^
3-methyl-1-butanol	243 ^a^	184 ^a^	233 ^a^	189 ^a^
acetic acid	16.5 ^a^	15.8 ^a^	14.7 ^a^	9.94 ^b^
acetoin	7.92 ^a^	8.53 ^a^	8.01 ^a^	7.00 ^a^
acetone	90.7 ^a^	73.2 ^a^	88.8 ^a^	56.6 ^a^
alpha-terpinene	2.92 ^a^	3.91 ^a^	2.96 ^a^	2.89 ^a^
ammonia	2542 ^a^	1278 ^b^	2874 ^a^	2973 ^a^
beta-caryophyllene	0.64 ^a^	1.47 ^a^	1.83 ^a^	1.07 ^a^
carbon disulfide	4130 ^a^	3723 ^ab^	3274 ^b^	3918 ^ab^
decanal	5.13 ^a^	6.32 ^a^	5.38 ^a^	5.07 ^a^
dimethyl disulfide	19,509 ^a^	18,579 ^ab^	16,006 ^b^	18,692 ^ab^
dimethyl sulfide	9.36 ^ab^	5.73 ^b^	13.14 ^a^	10.37 ^ab^
dimethylamine	771 ^b^	1012 ^a^	514 ^c^	536 ^c^
ethyl acetate	8.80 ^a^	9.48 ^a^	8.89 ^a^	7.78 ^a^
formaldehyde	1959 ^a^	1913 ^a^	1776 ^a^	1947 ^a^
geosmin	56.8 ^a^	53.1 ^ab^	40.6 ^b^	55.3 ^a^
heptanal	9.12 ^a^	10.5 ^a^	9.05 ^a^	9.35 ^a^
heptane	4881 ^a^	3320 ^a^	4426 ^a^	1970 ^a^
hexanal	44.4 ^a^	36.3 ^a^	58.1 ^a^	44.6 ^a^
hexanoic acid	2.83 ^ab^	3.11 ^a^	2.28 ^bc^	2.11 ^c^
hydrogen sulfide	2.03 ^a^	4.82 ^a^	4.67 ^a^	2.98 ^a^
indole	3.13 ^a^	1.80 ^a^	2.43 ^a^	2.80 ^a^
isobutyl alcohol	24,499 ^a^	23,342 ^ab^	20,703 ^b^	23,640 ^ab^
methyl mercaptan	23,259 ^a^	22,004 ^ab^	20,153 ^b^	22,644 ^ab^
nonanal	51.1 ^ab^	52.6 ^a^	42.9 ^b^	52.2 ^a^
oct-2-en-1-ol	2216 ^a^	2164 ^a^	1653 ^b^	1986 ^ab^
octanal	9.11 ^a^	9.81 ^a^	8.81 ^a^	8.49 ^a^
octane	144 ^a^	128 ^ab^	108 ^b^	140 ^ab^
pentanal	171 ^a^	116 ^ab^	155 ^ab^	70.2 ^b^
pentane	7869 ^a^	6282 ^b^	6525 ^ab^	6270 ^b^
propanal	130 ^a^	91.1 ^a^	148 ^a^	150 ^a^
trans-2-undecenal	34.3 ^a^	27.6 ^a^	26.9 ^a^	28.3 ^a^
trimethylamine	119 ^a^	88.7 ^a^	108 ^a^	96.9 ^a^
trimethylpyrazine	79.4 ^a^	74.8 ^a^	55.7 ^b^	71.2 ^ab^
